# Anthracene Bisureas as Powerful and Accessible Anion Carriers

**DOI:** 10.1002/chem.201800508

**Published:** 2018-04-06

**Authors:** Christopher M. Dias, Hennie Valkenier, Anthony P. Davis

**Affiliations:** ^1^ School of Chemistry University of Bristol Cantock's Close Bristol BS8 1TS UK; ^2^ Université Libre de Bruxelles Avenue F.D. Roosevelt 50, CP165/64 1050 Brussels Belgium

**Keywords:** anion transport, membranes, receptors, supramolecular chemistry, ureas

## Abstract

Synthetic anion carriers (anionophores) have potential as biomedical research tools and as treatments for conditions arising from defective natural transport systems (notably cystic fibrosis). Highly active anionophores that are readily accessible and easily deliverable are especially valuable. Previous work has resulted in steroid and *trans*‐decalin based anionophores with exceptional activity for chloride/nitrate exchange in vesicles, but poor accessibility and deliverability. This work shows that anthracene 1,8‐bisureas can fulfil all three criteria. In particular, a bis‐nitrophenyl derivative is prepared in two steps from commercial starting materials, yet shows comparable transport activity to the best currently known. Moreover, unlike earlier highly active systems, it does not need to be preincorporated in test vesicles but can be introduced subsequent to vesicle formation. This transporter also shows the ability to transfer between vesicles, and is therefore uniquely effective for anion transport at low transporter loadings. The results suggest that anthracene bisureas are promising candidates for application in biological research and medicine.

## Introduction

The transport of anions across biological membranes is essential for the proper functioning of a cell.^1^, ^2^ Anion concentration gradients play an important role in helping to regulate cell pH, maintaining cell volume, and electrical signaling.^1^, ^2^, ^3^ These gradients are maintained and controlled through the action of membrane‐bound proteins, which provide pathways through the apolar phospholipid bilayer.^1^, ^3^, ^4^, ^5^ Dysfunction of these proteins is known to give rise to a number of diseases including Bartter syndrome,^6^, ^7^ Dent's disease,^8^ and cystic fibrosis.^9^, ^10^ Synthetic anion transporters (anionophores) that can mimic the function of endogenous ion transport proteins could potentially be used to treat these conditions and their development has become an active area of supramolecular chemistry.^4^, ^11^, ^12^

**Figure 1:**
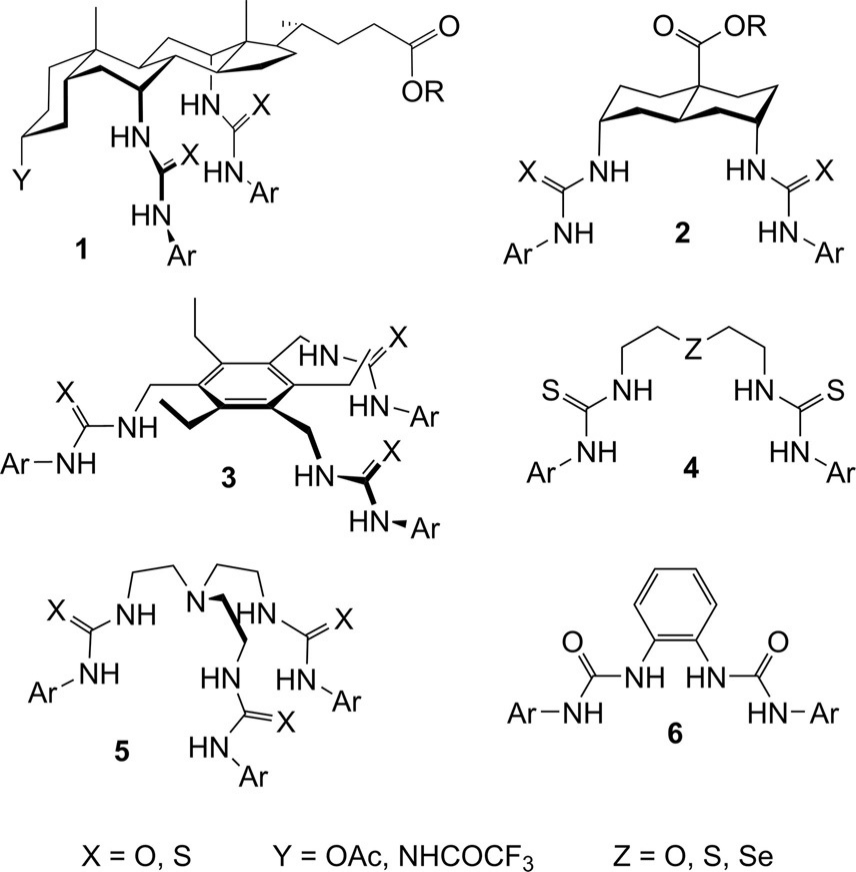
Scaffolds upon which powerful anionophores have previously been developed.

**Figure 2:**
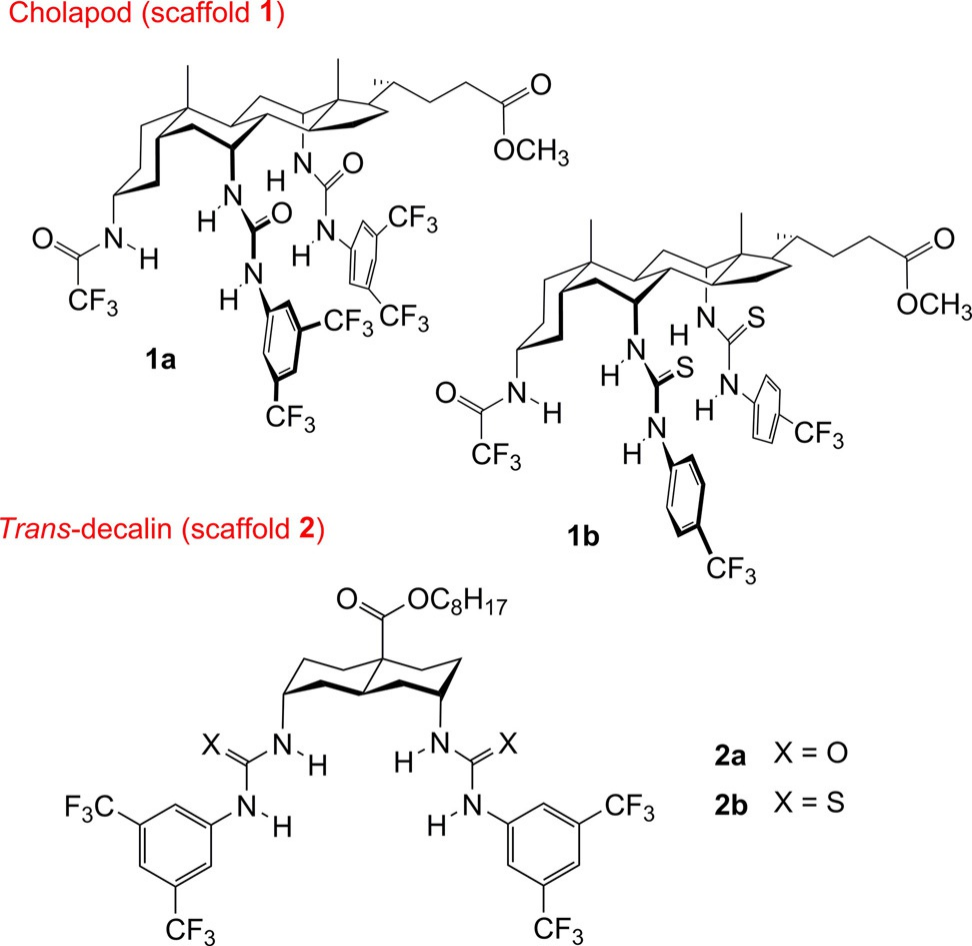
Structures of previously reported anionophores based on cholapod (**1**) and *trans*‐decalin (**2**) scaffolds.

An important goal in this area is the development of anion carriers that are (a) highly active, (b) easily synthesized, and (c) readily delivered to bilayer membranes. We have shown that ureas and thioureas in the cholapod (**1**)^13^, ^14^, ^15^, ^16^, ^17^, ^18^, ^19^ and *trans*‐decalin (**2**)^15^, ^17^, ^20^, ^21^, ^22^ series (Figure 1) can serve as highly effective chloride transporters in large unilamellar vesicles (LUVs). The most powerful, such as bisthioureas **1 b** and **2 b** (Figure 2), can promote significant Cl^−^/NO_3_^−^ exchange when present as single molecules in LUV membranes.^15^ *Trans*‐decalin **2 b**, the most active reported to date, transports 850 Cl^−^ ions per second and is comparable to a protein channel after allowing for molecular weight.^15^ However, while these carriers show high intrinsic activities, both families require quite lengthy syntheses. Moreover, the most active variants tend to be highly lipophilic, and do not transfer well to a preformed membrane. To express their activity, it is generally necessary to incorporate them in the bilayer during membrane production. Meanwhile other, simpler scaffolds also yield powerful anionophores (e.g., **3**–**6**,^23^, ^24^, ^25^, ^26^, ^27^, ^28^, ^50^ Figure 1), but none seem able to match the activities of the cholapod and *trans*‐decalin families.

**Figure 3:**
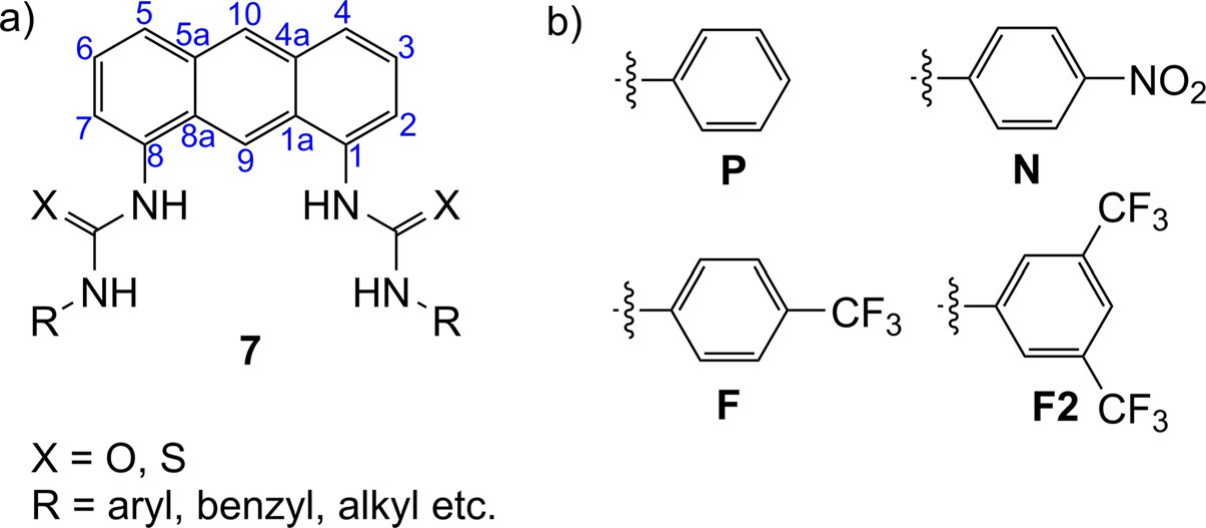
a) General structure **7** of anthracene 1,8‐bis(thio)ureas that have previously been applied as receptors. b) Terminal groups R used in this work. Molecules are labeled according to scaffold (**7**), X (**O**/**S**) and aromatic substituent (**P**/**N**/**F**/**F2**). For example, **7ON** refers to the bis‐*N*‐nitrophenylurea based on scaffold **7**.

A key feature of transporters **1** and **2** is the 1,5‐diaxial arrangement of (thio)urea binding units. Parallel bonds between scaffold and (thio)urea position the latter so that all four NH can bind to Cl^−^ simultaneously. Restricted rotation about these C−N bonds ensures that intramolecular hydrogen bonding cannot occur. Considering alternative structures that might be easier to synthesize, we realized that the 1,8‐disubstituted anthracenes **7** (Figure 3) bear a close geometric resemblance to the 1,5‐diaxial systems. Compounds **7** have previously been shown to function as receptors for anions^29^, ^30^, ^31^, ^32^ and neutral guests,^33^ but anionophore activity has not been investigated. Here, we report that anthracene bisureas **7** (X=O) can serve as outstandingly effective anionophores, competitive with the best of the 1,5‐diaxial systems and superior in some respects. Given its accessibility, this system could be considered the method of choice for inducing rapid chloride transport in bilayer membranes.

## Results and Discussion

### Design and synthesis

**Figure 4:**
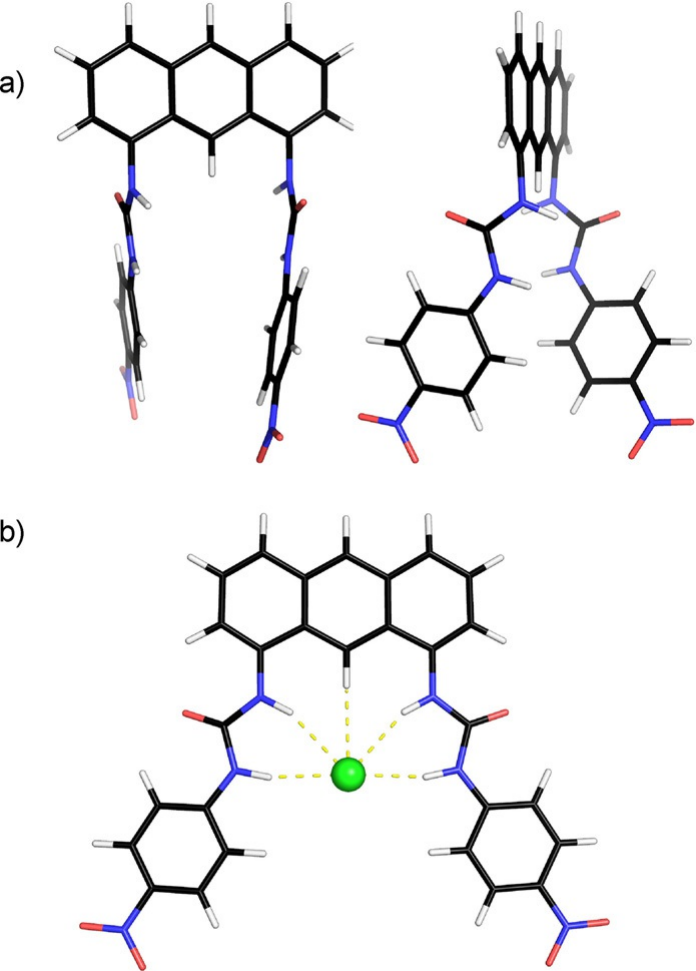
a) Calculated ground‐state structure of **7ON** (Monte Carlo Molecular Mechanics, followed by Hartree–Fock 6‐31G** optimization in Jaguar). b) Calculated structure of **7ON**⋅Cl^−^, employing similar methodology.^39^

Previous work has shown that electron‐withdrawing terminal groups favor binding and transport in (thio)urea‐based anionophores. 4‐Nitrophenyl **N**, 4‐trifluoromethylphenyl **F**, and 3,5‐bis(trifluoromethyl)phenyl (**F2**) (Figure 3) are especially well‐known and effective.^14^, ^15^, ^16^, ^17^, ^20^, ^23^, ^24^, ^25^, ^27^, ^28^, ^34^, ^35^ We therefore targeted anthracene‐based bisureas bearing these substituents (**7ON**, **7OF**, and **7OF2**; Figure 3), with unsubstituted bisurea **7OP** and the bisthiourea **7SF2** included for comparison purposes. Molecular modeling on **7ON** confirmed the potential for chloride binding and transport. The calculated ground state structure, shown in Figure 4 a, features roughly antiparallel urea groups. Although not preorganized for binding, they do not hydrogen bond to each other and are therefore free to rotate. On addition of chloride, the ureas adopt a convergent conformation to form a 1:1 complex, with H⋅⋅Cl distances of 2.3–2.5 Å (Figure 4 b). These distances are slightly shorter than the hydrogen bonds observed in a crystal structure of a decalin **2** with Cl^−^.^17^ The anthracenyl C(9)‐H atom is also positioned close to the chloride (H⋅⋅Cl=2.7 Å), potentially contributing to binding.^29^

**Scheme 1:**
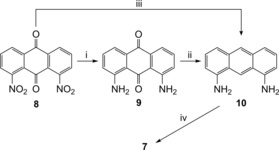
Synthesis of anthracene bis(thio)ureas: (i) Na_2_S⋅9 H_2_O, EtOH/H_2_O, reflux, 65 h, 98 %; (ii) NaBH_4_, NaOH, *i*PrOH, reflux, 16 h, 60 %; (iii) NaBH_4,_ *i*PrOH, reflux, 43 h, 31 %; (iv) **7O**(**P**/**N**/**F**/**F2**): ArNCO, CH_2_Cl_2_, reflux, 4–16 h, 50–90 %, **7SF2**: 3,5‐(CF_3_)_2_C_6_H_3_NCS, pyridine, RT, 21 h, 61 %.

The bis(thio)ureas were prepared by means of a short and straightforward process from the commercially available (and inexpensive) 1,8‐dinitroanthraquinone (**8**) (Scheme 1). Following literature procedures, **8** could be converted to diamine **10** by means of a single‐step reduction with NaBH_4_ 33 (31 % yield) or in two steps via diaminoquinone **9** (59 % overall yield).^29^, ^30^, ^32^ One further step is then required to generate the bis(thio)ureas, through treatment of **10** with the appropriate iso(thio)cyanate. Full synthetic procedures and characterization data are given in the Supporting Information.

### Binding studies

To underpin transport studies, the binding of **7** to chloride was characterized through titrations against Bu_4_N^+^Cl^−^ in [D_6_]DMSO/0.5 % H_2_O. Significant downfield shifts were observed for the (thio)urea N*H* and C(9)−H anthracenyl signals upon the addition of guest, which corroborates the computational prediction. To determine the stoichiometry of the host–guest complex(es) formed, we followed the approach outlined by Jurczak^36^ and Thordarson,^37^, ^38^ whereby titration data is fitted to all reasonable binding models (1:1, 1:2, and 2:1) and the residual distribution plots compared. The model which yields the lowest and most random distribution of residuals is the most likely to be valid.^39^ For bisureas **7OP**, **7ON**, **7OF**, and **7OF2**, a 1:1+2:1 (host:guest) binding stoichiometry was inferred and titration data were fitted to this model. Association constants calculated for the 1:1 complex were ≥2000 m ^−1^, significantly higher than that for decalin bisurea **2 a** (Table 1). This is remarkable when one compares the accessibility of the anthracene system with that of the decalins. The affinities are also higher than receptors based upon other simple scaffolds, for example, **3**–**6**, where binding constants measured under the same conditions do not typically exceed 10^3^ m ^−1^.^23^, ^24^, ^25^, ^26^, ^27^, ^28^, ^50^ The affinities are only modestly influenced by the electronic effects exerted by the terminal aryl groups, although unsubstituted bisurea **7OP** was found to be the weakest chloride receptor as anticipated. In the case of bisthiourea **7SF2**, analysis indicated much weaker binding, with *K*_a,1:1_=130 m ^−1^. This finding runs counter to normal expectation, given that thioureas are more acidic than ureas.^40^, ^41^, ^42^ However, calculations suggest that the bulk of the thiourea S might disfavor the near‐planar conformation required for strong binding.^39^ A similar phenomenon has previously been observed in receptors based on scaffold **6**, which are structurally related.^43^

**Table 1:** Binding and transport data for bis(thio)ureas in the cholapod (**1**), *trans*‐decalin (**2**), and anthracene (**7**) series.

Compound	*c* log*P* ^[a]^	Binding to Bu_4_N^+^Cl^−^ in [D_6_]DMSO/0.5 % H_2_O^[b]^		Chloride transport in LUVs		
		*K*_a, 1:1_ [M^−1^]	K_a, 2:1_ [M^−1^]	*t* ${}_{1/2}$ [s]^[e]^	[*I*] [s^−1^]^[f]^	*D* ^[i]^
**1 a** 15, 17	12.0	n.d.	–	64	450	0.12
**1 b** 15, 17	10.2	17 000^[c]^	–	15	1800	0.37
**2 a** 15	11.3	880^[c,j]^	–	88	340	n.d.
**2 b** 15, 45	11.6	2600^[c]^	–	9	3800	0.03
**7OP**	7.3	2000^[d]^	240^[d]^	277	90	1
**7ON**	6.9	2600^[d]^	210^[d]^	22	2100	0.82
**7OF**	9.3	2200^[d]^	250^[d]^	45	1200	0.62
**7OF2**	11.4	3000^[d]^	390^[d]^	30	1900	0.45
**7SF2**	11.7	130^[g]^	^[g]^	^[h]^	^[h]^	n.d.

n.d.=not determined. [a] Calculated log*P*, an estimate of lipophilicity. Values were calculated using TorchLite (available as freeware from http://www.cresset-group.com). [b] Obtained through ^1^H NMR titrations at 298 K. [c] Obtained by fitting all data points to a 1:1 binding model using a least‐square fitting procedure. [d] Obtained by fitting all data points to a 1:1+2:1 (receptor:chloride) binding model (Nelder–Mead method) using the Bindfit v0.5 applet (available as freeware from app.supramolecular.org). [e] Obtained by fitting *F*_0_/*F* versus time (0–500 s) to a single exponential decay function when transporter:lipid=1:25k. [f] Specific initial rate: initial slope for *F*_0_/*F* versus time, divided by transporter/lipid ratio and averaged for a range of experiments at different loadings. [g] Obtained by fitting all data points to a 1:1+1:2 binding model (method as for [d] above); *K*_a, 1:2_=14 m ^−1^. [h] Transport was observed (see Figure 5 a) but not quantified, due to the instability of **7SF2** in the medium.^39^ [i] *D*=deliverability. Calculated by dividing *I* for the external addition experiment by that observed when the anionophore was preincorporated. [j] Measured for the ethyl ester analogue of **2 a**. The ester side‐chain is not expected to affect affinities.^15^

### Anion transport

Anion transport by **7** was assessed using the previously reported lucigenin assay for Cl^−^/NO_3_^−^ exchange.^44^ Briefly, LUVs (ca. 200 nm diameter) containing aqueous NaNO_3_ (225 mm) and the halide‐sensitive dye lucigenin were prepared from 1‐palmitoyl‐2‐oleoyl‐*sn*‐glycero‐3‐phosphocholine (POPC) and cholesterol (7:3 ratio) with the test anionophore preincorporated in the membrane. The LUVs were suspended in aqueous NaNO_3_ (225 mm) and placed inside a fluorescence spectrometer. The experiment was commenced by the addition of NaCl (25 mm) and the influx of chloride monitored by the decay in lucigenin fluorescence.^39^

Transport rates were quantified by fitting the inverse of the normalized fluorescence traces (*F*_0_/*F*) to single exponential function to obtain approximate half‐lives (*t* ${}_{1/2}$ ) and a double exponential function to obtain initial rates (*I*). Dividing *I* by the transporter/lipid ratio and averaging for a range of experiments at different loadings gives the specific initial rate, [*I*]. As described in previous work,^15^ [*I*] is independent of the transporter to lipid loading and thus allows the performance of anionophores with widely different activities to be compared directly.

Results from these experiments are summarized in Table 1 and Figure 5. All the anthracene bisureas were found to mediate chloride transport, with activities measurable at transporter to lipid loadings as low as 1:250k (1.6 nm overall in the aqueous suspension) (Figure 5 b). Consistent with previous observations,^15^, ^16^, ^20^, ^25^, ^28^, ^34^, ^35^ the electron‐deficient aryl termini **N**, **F**, and **F2** promoted faster transport than unsubstituted **P**. The most powerful variant was the nitrophenyl bisurea **7ON**, for which [*I*] was measured as 2100 s^−1^. A dose‐response study employing six different loadings of **7ON** (Figure 6 a) revealed that this compound was significantly active even at the lowest transporter to lipid ratio of 1:1000k. At this loading most LUVs contain either 1 or 0 transporter molecules, thus the activity observed corresponds to **7ON** acting as a single molecule.^15^

**Figure 5:**
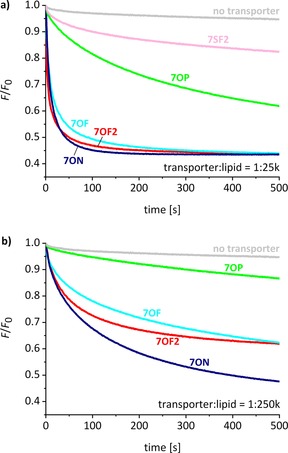
Chloride transport mediated by anthracene‐based bis(thio)ureas in 200 nm POPC/cholesterol (7:3) LUVs as measured using the lucigenin method. The anionophore was preincorporated in the membrane at a transporter to lipid loading of a) 1:25k or b) 1:250k.

**Figure 6:**
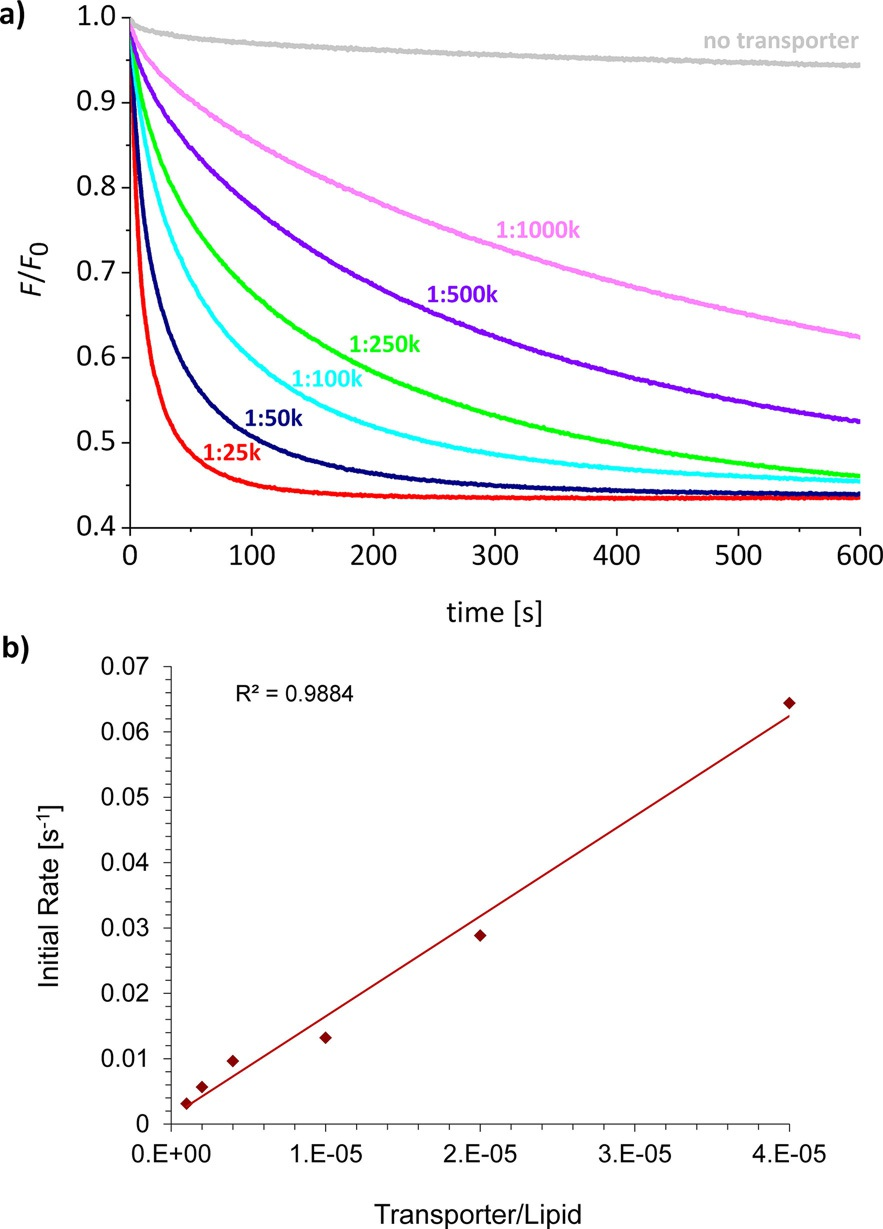
Chloride transport mediated by **7ON** in 200 nm POPC/cholesterol (7:3) LUVs when preincorporated at various transporter to lipid loadings. (a) Traces for *F*/*F*_0_ versus time. (b) Plot of the initial rate of transport induced by **7ON** as a function of its concentration inside LUVs.

**Figure 7:**
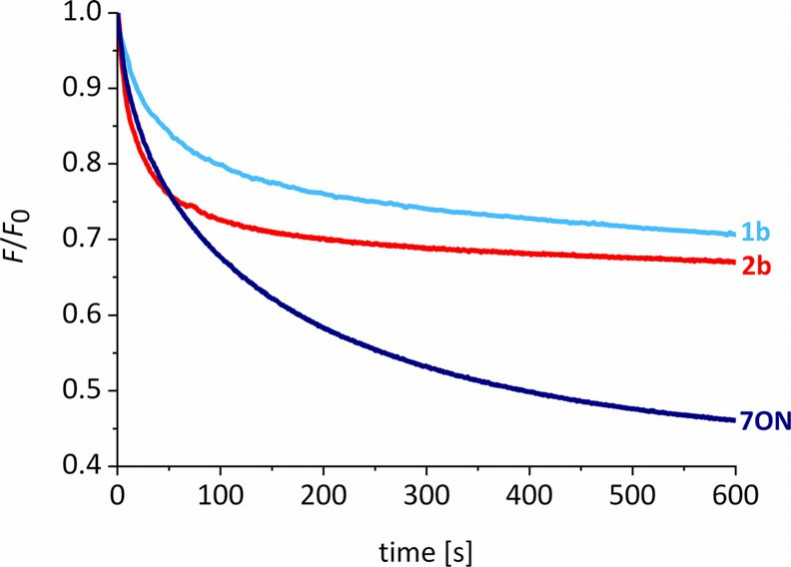
Chloride transport mediated by compounds **1 b**, **2 b**, and **7ON** in 200 nm POPC/cholesterol (7:3) LUVs as measured using the lucigenin method. The anionophore was preincorporated in the membrane at a transporter to lipid loading of 1:250k in all cases.

Comparing **7ON** to earlier systems, the anthracene does not quite match decalin **2 b**, the current record‐holder at [*I*]=3800 s^−1^. However, the examination of fluorescence decay traces shows that in some respects the new system is more effective. We have previously found that powerful transporters such as **2 b** and **1 b** produce rapid initial drops in emission at the low loadings, but that traces tend to plateau at relatively high levels. This is illustrated in Figure 7 for transporter:lipid=1:250k. We believe this is due to the absence of transporter molecules from many vesicles (see above), which therefore act as bystanders. In contrast, the traces for **7ON** tend towards lower emission values at all loadings (Figures 6 a and 7). Thus, as the experiment progresses, the overall effect of **7ON** becomes greater than any of the earlier systems. The reason for the difference is thought to be the ability of **7ON** to transfer between vesicles, unlike **2 b**, which is trapped in its original location. Experiments supporting this hypothesis are described in the next section.

Considering their accessibility, the effectiveness of the anthracene bisureas is remarkable. In contrast to decalin **2 b**, which requires a nine‐step synthesis,^15^ the anthracenes are available in just two steps. Moreover, it is notable that **7ON** and **7OF2** are ureas, whereas **2 b** possesses the more favorable thiourea units. Decalin bisurea **2 a** is considerably less active than the anthracene bisureas, suggesting that the anthracene scaffold is more effective than the decalin. It is more difficult to compare the transporters with systems from other groups, but the dose‐response data for **7ON** (Figure 6) allows the estimation of an EC_50, 270 s_,^46^ a measure which is widely used by other laboratories. The EC_50, 270 s_ value calculated for **7ON** is 0.0003 mol %, the lowest reported to date for chloride–nitrate exchange, and even lower than that of the natural anionophore prodigiosin.^28^

In contrast to the ureas, and counter to trends observed previously,^15^ the bisthiourea **7SF2** proved relatively ineffective (Figure 5 a). While its modest chloride affinity may be a factor, it also showed limited stability under the conditions of the transport experiment.^39^ The anthracene bisthiourea design was therefore not pursued further.

### Mechanistic studies

Although the anthracene bisureas were designed as anion carriers, it is also possible that they could act through formation of self‐assembled channels. The linear relationship between initial transport rates and transporter:lipid ratios for **7ON** (Figure 6 b) provides one line of evidence for the carrier mechanism; if more than one transporter molecule is required to form the active complex, one would normally expect reduced effectiveness at lower concentrations.^47^ To support this conclusion, transport was also studied in vesicles composed of 1,2‐dipalmitoyl‐*sn*‐glycero‐3‐phosphocholine (DPPC), which undergoes a transition between gel and liquid phases at 41 °C.^48^ The transition is expected to affect transport rates for mobile carriers, but much less so for channels. As anticipated, **7ON** proved inactive at 25 °C (gel phase), but active at 45 °C (liquid phase), consistent with the carrier mechanism.^39^, ^49^

Studies were also undertaken to test the ability of the transporters to move between vesicles, as implied for **7ON** by the results discussed earlier. “Delivery vesicles” containing **7ON** but not lucigenin were mixed with “receiver vesicles” containing lucigenin but not **7ON**, before addition of chloride. Fluorescence decay traces implied that the transporter was transferred rapidly to the receiver vesicles, equilibration occurring in ≤5 min.^39^ In contrast, the same experiments with the more lipophilic **7OF2** showed negligible transfer on the same timescale. Despite the ability of **7ON** to exchange between vesicles, experiments designed to detect leaching from the membranes gave negative results, implying that the equilibrium concentration in water is very low.^39^

### Deliverability

**Figure 8:**
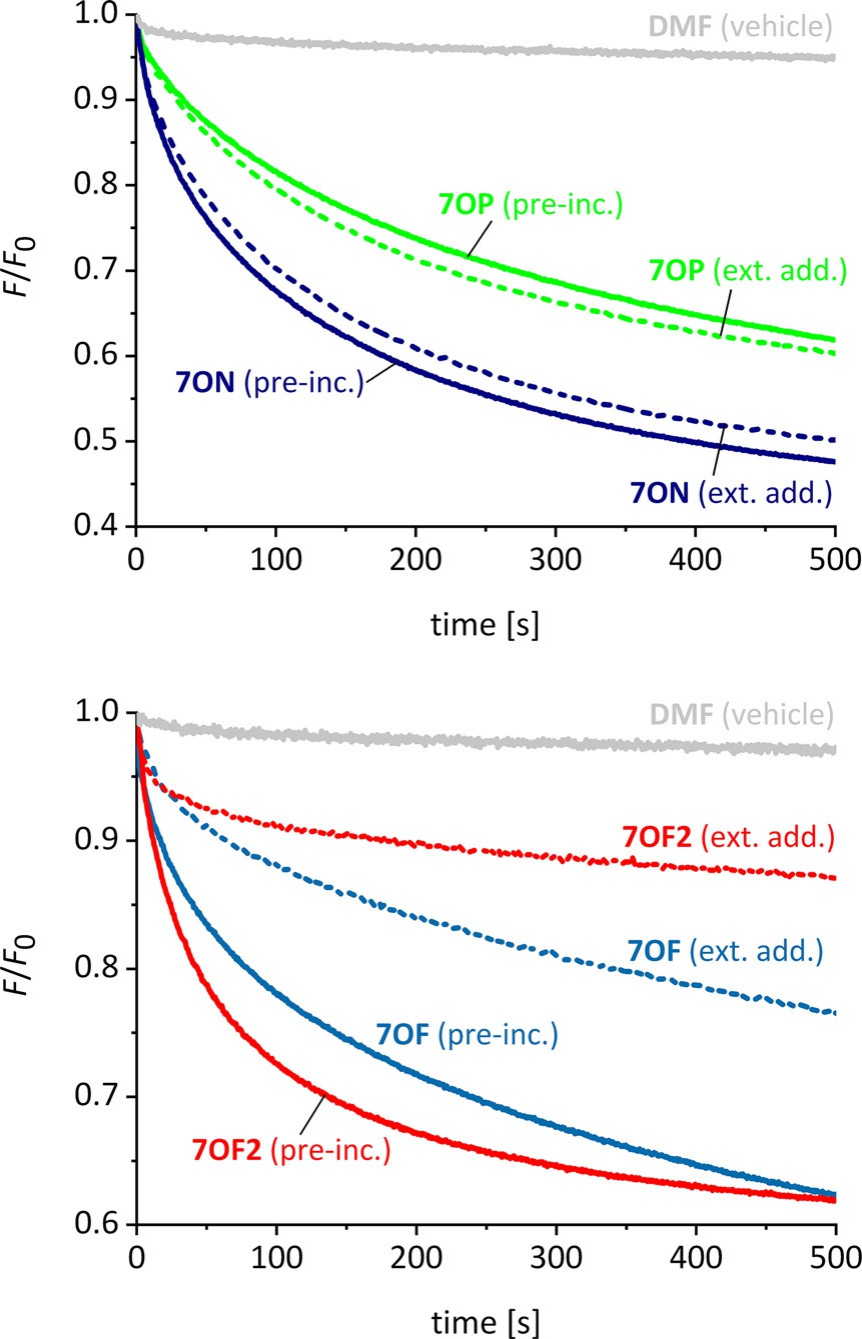
Chloride transport mediated by anthracene bisureas when the anionophore was either preincorporated in the membrane (solid lines) or added externally as solution in DMF to vesicles without anionophore (dashed lines). The notional transporter to lipid loading was 1:25k for **7OP** and 1:250k for **7ON**, **7OF**, and **7OF2**.

An important requirement for practical applications of anionophores is that they must be readily deliverable to target membranes. The most active cholapod and *trans*‐decalin carriers do not fulfil this criterion well. Decalin **2 b**, in particular, is almost inactive when added to preformed LUVs and is only effective when incorporated in the vesicles as they are prepared.^15^, ^45^ To provide a quantitative estimate of deliverability, we have developed a variant of the lucigenin assay in which the vesicles are formed without transporter, and the latter is then added using a standardized procedure, before the introduction of chloride.^17^ The decay of *F*_0_/*F* is followed, and the initial rate *I* is measured. Deliverability (*D*) is quantified by dividing *I* for this experiment by that observed when the anionophore was preincorporated. Fluorescence decay traces for both types of experiment, applied to the four anthracene bisureas, are shown in Figure 8. Values of *D* for the anthracene bisureas, as well as **1 a**, **1 b**, and **2 b**, are listed in Table 1. The results show that deliverability for **7OF** and **7OF2** is only moderate, but that **7OP** and **7ON** are transferred quite efficiently to the vesicles. In particular, the deliverability of **7ON**, at *D*=0.82, contrasts starkly with that of **2 b** (*D*=0.03). The good deliverability of **7ON** probably relates to its moderate lipophilicity (*c* log *P*=6.9, see Table 1). We suspect that highly lipophilic agents such as **2 b** (*c* log *P*=11.6) form intractable aggregates after addition to the aqueous phase, and these interact poorly with the membranes. Though **7ON** presumably also aggregates, the individual molecules are less lipophilic and this could lead to improved availability.

## Conclusion

In conclusion, we have shown that anthracene 1,8‐bisureas are exceptionally effective and practical anion transporters. The most powerful promote chloride/nitrate exchange at rates comparable to the highest previously observed, while being far more accessible than the earlier systems. Dinitro variant **7ON** combines high activity with good deliverability in a manner unmatched by previous systems. Taking into account its ability to transfer between vesicles, it is arguably the most effective agent currently available for transporting chloride across vesicle membranes at low dosages. The anthracene scaffold has potential for further modification to control binding affinities, lipophilicities etc. We believe the design has promise for application in tools for biomedical research, and perhaps in the treatment of channelopathies such as cystic fibrosis.

## Conflict of interest

The authors declare no conflict of interest.

## Supporting information

As a service to our authors and readers, this journal provides supporting information supplied by the authors. Such materials are peer reviewed and may be re‐organized for online delivery, but are not copy‐edited or typeset. Technical support issues arising from supporting information (other than missing files) should be addressed to the authors.

Supplementary
