# Supplementary material for: Anthracene Bisureas as Powerful and Accessible Anion Carriers
Source: Chemistry. 2018 Apr 6;24(23):6262–8. doi: 10.1002/chem.201800508 (PMC5947650; doi:10.1002/chem.201800508)
Supplement: Supplementary file 1 — Supplementary [file CHEM-24-6262-s001.pdf]

CHEMISTRY
A European Journal

Supporting Information

Anthracene Bisureas as Powerful and Accessible Anion Carriers

Christopher M. Dias,[a] Hennie Valkenier,*[b] and Anthony P. Davis*[a]

chem_201800508_sm_miscellaneous_information.pdf

Contents

1. Molecular Modelling 3

2. Synthesis and Characterisation 4

2.1. General Considerations 4

2.2. Synthetic Procedures 4

2.3. NMR Spectra 10

3. Determination of Chloride Binding Affinities through ^{1}H NMR Titrations 17

3.1. Method 17

3.2. NMR Stackplots, Binding Curves and Residual Distribution Plots 18

4. Transport Studies 28

4.1. General Considerations 28

4.2. The Lucigenin Method 28

4.3. Quantification of Transport Rates 29

4.4. Hill Analysis on Bisurea **7ON** 30

4.5. Deliverability Studies 30

4.6. Transport Mediated by **7ON** in DPPC Vesicles 31

4.7. Exchange of Bisurea **7ON** between Vesicles 33

4.8. Exchange of Bisurea **7OF2** between Vesicles 34

4.9. Leaching Studies on Bisurea **7ON** 35

5. Decomposition Studies 37

6. References 39

1. Molecular Modelling

All modelling was performed using Maestro 11.2.014 (Schrödinger Inc.).

*Anthracene bisurea **7ON***

A Monte Carlo Molecular Mechanics (MCMM) conformational search on **7ON** was performed in MacroModel, employing the MMFFs force-field in $CHCl_3$, giving a ground state in which the urea groups are only slightly twisted out of the plane of the anthracene. This conformation was then energy minimised in Jaguar (Hartree-Fock, 6-31G**), and the result is given in Figure 4a of the main text.

*Chloride complex of **7ON***

A MCMM conformational search on the chloride complex of **7ON** was performed in MacroModel, employing the MMFFs force-field in vacuum. The resulting ground-state conformation was then energy minimised in Jaguar (Hartree-Fock, 6-31G**), and the resulting structure is shown in Figure 4b of the main text. In this structure, NH···Cl^- distances are 2.312, 2.332, 2.474 and 2.497 Å, and the C(9)H···Cl^- distance is 2.703 Å.

*Comparison of **7SN** and **7ON***

Starting from the nearly flat complex **7ON.Cl$^-$**, the chloride was removed from the structure and the two oxygen atoms were replaced by sulfur atoms to obtain structure **7SN**. This was energy minimised in MacroModel, using the MMFFs force-field in vacuum. The results are presented in Figure S1, along with those of **7ON** treated identically for comparison.

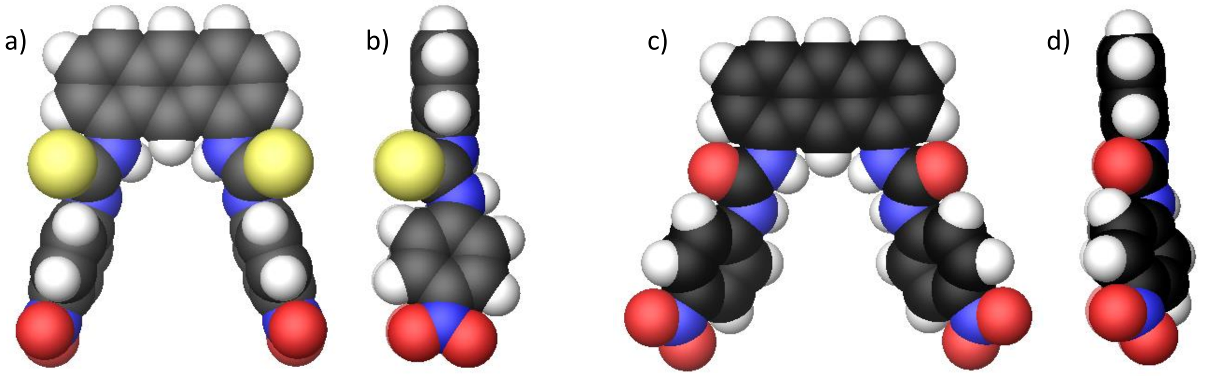

Figure S1. Comparison of the structures of **7SN** (a, b) and **7ON** (c, d) upon energy minimisation in MacroModel (MMFFs), when starting from flat structures. These models show that the large sizes of the sulfur atoms prevent **7SN** from adopting a flat conformation, while **7ON** can adopt a nearly flat conformation.

2. Synthesis and Characterisation

2.1. General Considerations

All commercially available materials were used without further purification unless otherwise stated. DCM was dried by passing through a modified Grubbs system[1] manufactured by Anhydrous Engineering. Pyridine was dried with CaH_2 followed by vacuum distillation. Reactions were monitored by TLC using Merck silica gel 60 F_{254} TLC plates. Spots were visualised under visible or ultraviolet light (254/365 nm). Flash column chromatography was performed on silica gel (technical grade, 40–63 µm particle size).

^{1}H, ^{13}C and ^{19}F NMR spectra were recorded on Bruker Avance III HD 500 (carbon sensitive), Varian VNMRS 500a (carbon sensitive), Varian VNMRS 500b (proton sensitive) or JEOL ECS 400 spectrometers. Chemical shifts (δ/ppm) are referenced relative to residual solvent signals in the deuterated solvent. Data are reported as follows: s = singlet, d = doublet, dd = doublet of doublets, t = triplet, q = quartet, br = broad.

Infrared (IR) spectra were recorded on a PerkinElmer Spectrum Two FT-IR spectrometer, with signal intensities reported as follows: w = weak, m = medium, s = strong, br = broad.

Mass spectrometry was performed on a Bruker micrOTOF II spectrometer using electrospray ionisation (positive ion mode) or on a Bruker ultrafleXtreme spectrometer using MALDI.

2.2. Synthetic Procedures

*1,8-diaminoanthraquinone (**9**)*

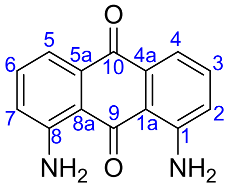

This compound was prepared according to a literature procedure[2] with minor modifications. To a suspension of 1,8-dinitroanthraquinone (**8**, 2 g, 6.71 mmol) in EtOH (40 mL) was added a solution of $Na_2S{\cdot}9H_2O$ (6.41 g, 26.7 mmol) in H_2O (134 mL) and the resultant green suspension refluxed for 64.5 h. The reaction mixture was allowed to cool to r.t. and then poured into an ice/water mixture (134 mL) with stirring. The resultant precipitate was filtered, washed with ice-cold H_2O (2 x 20 mL) and dried under high vacuum to give the title compound as a red/purple solid (1.56 g, 98%). R_f 0.79 (4:1 EtOAc−hexane). ^{1}H NMR (500 MHz, $CDCl_3$) δ 7.62 (dd, *J* = 7.4, 1.2 Hz, 2H, **H4** & **H5**), 7.41 (dd, *J* = 8.3, 7.4 Hz, 2H, **H3** & **H6**), 6.94 (dd, *J* = 8.3, 1.2 Hz, 2H, **H2** & **H7**). ^{13}C NMR (126 MHz, $CDCl_3$) δ 189.1 (**C9**),

184.3 (**C10**), 150.7 (**C1** & **C8**), 134.5 (**C4a** & **C5a**), 134.0 (**C3** & **C6**), 123.3 (**C2** & **C7**), 117.0 (**C4** & **C5**), 115.2 (**C1a** & **C8a**). IR (neat) v/cm^{-1} 3472 (m, N–H), 3436 (m, N–H), 3332 (m, N–H), 3308 (m, N–H), 1655 (m), 1631 (m), 1592 (s), 1529 (s), 1451 (m), 1266 (s), 1167 (m). HRMS (ESI) *m/z* calcd for $C_{14}H_{10}N_2NaO_2$ [M+Na]$^+$ 261.0634, found 261.0645.

*1,8-diaminoanthracene (**10**)*

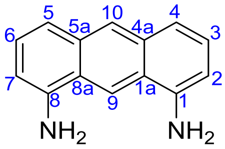

This compound was prepared according to a literature procedure[2] with minor modifications. To a two-neck round-bottom flask was added NaOH (17 mg, 0.43 mmol), 1,8-diaminoanthraquinone (**9**, 469 mg, 1.97 mmol) and *i*-PrOH (17 mL). The flask was evacuated and backfilled with N_2 (x3) and the solution purged with N_2 for 45 min. $NaBH_4$ (810 mg, 21.4 mmol) was added portionwise under N_2 and the resultant purple suspension left to reflux for 15.5 h. After this time an orange precipitate had formed. The reaction mixture was allowed to cool to r.t. and then poured into an ice/water mixture (50 mL) with stirring. The resultant precipitate was filtered, washed with ice-cold H_2O (2 x 10 mL) and dried under high vacuum to give a brown solid. This was purified by flash column chromatography (SiO_2; 3→10% MeOH in $CHCl_3$) to give the title compound as a gold/brown solid (246 mg, 60%). R_f 0.51 (1:9 MeOH–$CHCl_3$). ^{1}H NMR (500 MHz, $CDCl_3$) δ 8.37 (s, 1H, **H9**), 8.35 (s, 1H, **H10**), 7.49 (d, *J* = 8.5 Hz, 2H, **H4** & **H5**), 7.29 (dd, *J* = 8.5, 7.1 Hz, 2H, **H3** & **H6**), 6.76 (d, *J* = 7.1 Hz, 2H, **H2** & **H7**), 4.30 (br s, 4H, N**H**$_2$). ^{13}C NMR (126 MHz, $CDCl_3$) δ 142.1 (**C1** & **C8**), 132.6 (**C4a** & **C5a**), 127.3 (**C10**), 126.1 (**C3** & **C6**), 123.2 (**C1a** & **C8a**), 119.5 (**C4** & **C5**), 113.0 (**C9**), 108.1 (**C2** & **C7**). IR (neat) v/cm^{-1} 3423 (w, N–H), 3339 (w, N–H), 3228 (w, N–H), 3046 (w), 3006 (w), 1623 (m), 1559 (m), 1456 (m), 1400 (m), 1329 (m). HRMS (ESI) *m/z* calcd for $C_{14}H_{13}N_2$ [M+H]$^+$ 209.1073, found 209.1075.

*Alternative synthesis from 1,8-dinitroanthraquinone (**8**)*

This alternative synthesis is based on that reported in Ref. [3] with minor modifications. To a two-neck round-bottom flask was added 1,8-dinitroanthraquinone (**8**, 500 mg, 1.68 mmol) and *i*-PrOH (20 mL) to give a yellow suspension. The flask was evacuated and backfilled with N_2 (x3) and the suspension purged with N_2 for 2 h. $NaBH_4$ (763 mg, 20.2 mmol) was then added portionwise to the reaction mixture and the resultant dark green solution left to reflux under N_2 for 27 h. After this time, additional $NaBH_4$ (380 mg, 10.0 mmol) was added and the resultant dark brown solution left to reflux for a further 16 h. The solution was allowed to cool to r.t. and then poured into an ice/water mixture (60 mL) with stirring. The resultant precipitate was filtered, washed with ice cold H_2O (2 x 10 mL) and dried under

high vacuum to give a brown solid (330 mg). This was purified by flash column chromatography (SiO_2; 0→3% MeOH in $CHCl_3$) to give the title compound as a dark brown/yellow solid (108 mg, 31%).

*1,1'-(anthracene-1,8-diyl)bis(3-phenylurea) (**7OP**)*

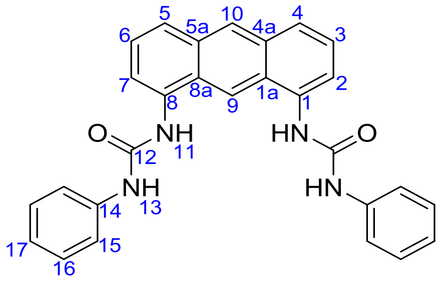

This compound was prepared according to a literature procedure[4] with minor modifications. To a solution of 1,8-diaminoanthracene (**10**, 30 mg, 0.14 mmol) in anhydrous DCM (3 mL) was added phenyl isocyanate (63 µL, 0.58 mmol) and the resultant brown suspension left to reflux under N_2 for 16 h. The reaction mixture was allowed to cool to r.t. and the resultant precipitate filtered, washed with $CHCl_3$ (x10) and dried under high vacuum to give a dark yellow solid (48 mg). This was further washed with acetone (x10) and dried under high vacuum to give the title compound as a yellow solid (32 mg, 50%). R_f 0.70 (1:9 MeOH–$CHCl_3$). ^{1}H NMR (500 MHz, DMSO-d_6) δ 9.09 (s, 2H, **H13**), 9.02 (s, 2H, **H11**), 8.92 (s, 1H, **H9**), 8.62 (s, 1H, **H10**), 7.92 (d, *J* = 7.3 Hz, 2H, **H2** & **H7**), 7.84 (d, *J* = 8.5 Hz, 2H, **H4** & **H5**), 7.55 (d, *J* = 7.7 Hz, 4H, **H15**), 7.52 (t, 7.9 Hz, 2H, **H3** & **H6**), 7.32 (t, 7.9 Hz, 4H, **H16**), 7.01 (t, *J* = 7.4, 2H, **H17**). ^{13}C NMR (126 MHz, DMSO-d_6) δ 153.0 (**C12**), 139.7 (**C14**), 134.2 (**C1** & **C8**), 131.7 (**C4a** & **C5a**), 128.9 (**C16**), 127.2 (**C10**), 125.8 (**C3** & **C6**), 125.2 (**C1a** & **C8a**), 123.0 (**C4** & **C5**), 121.9 (**C17**), 118.2 (**C15**), 117.0 (**C2** & **C7**), 114.0 (**C9**). IR (neat) *v*/cm^{-1} 3675 (br w), 3278 (br w, N–H), 2988 (s, C–H), 2976 (s, C–H), 2901 (s, C–H), 1640 (m), 1557 (m), 1394 (m), 1249 (m), 1227 (m), 1066 (s). HRMS (ESI) *m/z* calcd for $C_{28}H_{22}N_4NaO_2$ $[M+Na]^+$ 469.1635, found 469.1648.

*1,1'-(anthracene-1,8-diyl)bis(3-(4-nitrophenyl)urea) (**7ON**)*

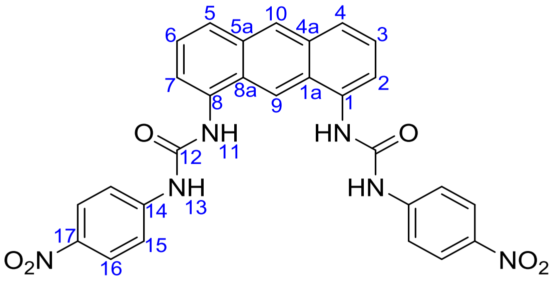

This compound was prepared according to a literature procedure[4] with minor modifications. To a 10 mL round bottom flask was added 1,8-diaminoanthracene (**10**, 23 mg, 0.11 mmol) and 4-nitrophenyl isocyanate* (76 mg, 0.46 mmol). Anhydrous DCM (3 mL) was added and the resultant suspension refluxed under N_2 for 3.5 h. The reaction mixture was allowed to cool to r.t. and the resultant precipitate filtered, washed with $CHCl_3$ (x10) and dried under high vacuum to give the title compound as a yellow solid (52 mg, 88%). ^{1}H NMR (400 MHz, DMSO-d_6) δ 9.75 (s, 2H, **H13**), 9.16 (s, 2H, **H11**), 8.87 (s, 1H, **H9**), 8.68 (s, 1H, **H10**), 8.19 (d, *J* = 9.2 Hz, 4H, **H16**), 7.93 (d, *J* = 8.5 Hz, 2H, **H4** & **H5**), 7.83 (dd, *J* = 7.3, 1.0 Hz, 2H, **H2** & **H7**), 7.75 (d, *J* = 9.2 Hz, 4H, **H15**), 7.55 (dd, *J* = 8.5, 7.3 Hz, 2H, **H3** & **H6**). ^{13}C NMR (126 MHz, DMSO-d_6) δ 152.7 (**C12**), 146.4 (**C14**), 141.0 (**C17**), 133.5 (**C1** & **C8**), 131.8 (**C4a** & **C5a**), 127.3 (**C10**), 125.9 (**C1a** & **C8a**), 125.7 (**C3** & **C6**), 125.2 (**C16**), 124.3 (**C4** & **C5**), 118.9 (**C2** & **C7**), 117.5 (**C15**), 114.7 (**C9**). IR (neat) *v*/cm^{-1} 3675 (br w), 3286 (br w, N–H), 2988 (s, C–H), 2973 (s, C–H), 2901 (s, C–H), 1646 (m), 1568 (m), 1505 (m), 1348 (m), 1066 (s). HRMS (MALDI) *m/z* calcd for $C_{28}H_{20}N_6NaO_6$ $[M+Na]^+$ 559.1337, found 559.1331.

*1,1'-(anthracene-1,8-diyl)bis(3-(4-(trifluoromethyl)phenyl)urea) (**7OF**)*

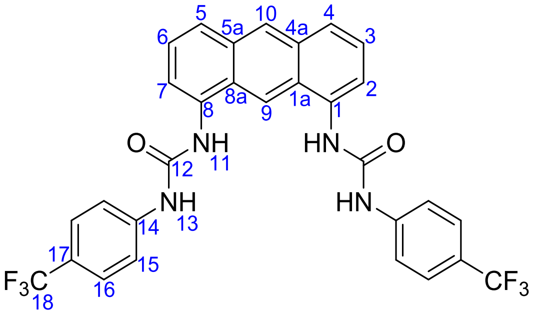

To a solution of 1,8-diaminoanthracene (**10**, 23 mg, 0.11 mmol) in anhydrous DCM (3 mL) was added 4-(trifluoromethyl)phenyl isocyanate (66 µL, 0.46 mmol) and the resultant brown suspension left to reflux under N_2 for 4.5 h. The reaction mixture was allowed to cool to r.t. and the resultant precipitate

* The isocyanate was purified before use: 3 mL of anhydrous DCM was added to the crude isocyanate (~300 mg). The undissolved bright yellow solid was removed from the solution by filtering through a cotton wool plug. The filtrate was concentrated *in vacuo* to give a pale yellow solid (~200 mg) which was used in this reaction.

filtered, washed with DCM (x10) and dried under high vacuum to give the title compound as a yellow/brown solid (58 mg, 90%). R_f 0.61 (1:9 MeOH–$CHCl_3$). ^1H NMR (400 MHz, DMSO-d_6) δ 9.46 (s, 2H, **H13**), 9.09 (s, 2H, **H11**), 8.90 (s, 1H, **H9**), 8.66 (s, 1H, **H10**), 7.89 (d, *J* = 8.7 Hz, 2H, **H4** & **H5**), 7.87 (dd, *J* = 7.4, 1.0 Hz, 2H, **H2** & **H7**), 7.73 (d, *J* = 8.5 Hz, 4H, **H15**), 7.64 (d, *J* = 8.6 Hz, 4H, **H16**), 7.54 (dd, *J* = 8.5, 7.4 Hz, 2H, **H3** & **H6**). ^{13}C NMR (126 MHz, DMSO-d_6) δ 152.9 (**C12**), 143.5 (**C14**), 133.8 (**C1** & **C8**), 131.8 (**C4a** & **C5a**), 127.3 (**C10**), 126.2 (q, *J* = 3.7 Hz, **C16**), 125.7 (**C3** & **C6**), 125.6 (**C1a** & **C8a**), 124.6 (q, *J* = 270.7 Hz, **C18**), 123.8 (**C4** & **C5**), 121.8 (q, *J* = 32.1 Hz, **C17**), 118.1 (**C2** & **C7**), 117.9 (**C15**), 114.4 (**C9**). ^{19}F NMR (377 MHz, DMSO-d_6) δ -60.0 (18-$\mathbf{CF_3}$). IR (neat) *v*/cm^{-1} 3675 (br w), 3288 (br w, N–H), 2988 (m, C–H), 2972 (m, C–H), 2901 (m, C–H), 1645 (m), 1557 (m), 1326 (m), 1106 (s), 1068 (s). HRMS (MALDI) *m/z* calcd for $C_{30}H_{20}F_6N_4NaO_2$ $[M+Na]^+$ 605.1383, found 605.1378.

*1,1'-(anthracene-1,8-diyl)bis(3-(3,5-bis(trifluoromethyl)phenyl)urea) (**7OF2**)*

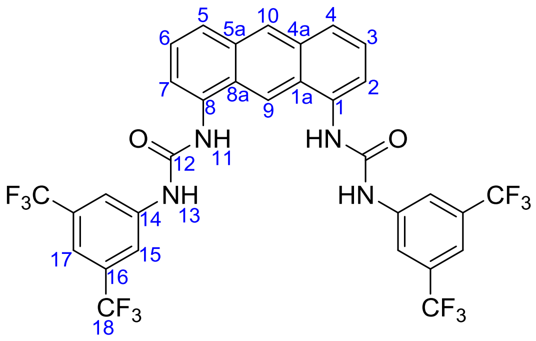

To a solution of 1,8-diaminoanthracene (**10**, 50 mg, 0.24 mmol) in anhydrous DCM (7 mL) was added 3,5-bis(trifluoromethyl)phenyl isocyanate (173 µL, 1.00 mmol) and the resultant dark brown suspension refluxed under N_2 for 3.5 h. The reaction mixture was allowed to cool to r.t. and the resultant precipitate filtered, washed with DCM (x10) and dried under high vacuum to give the title compound as a grey/brown solid (121 mg, 70%). R_f 0.23 (1:1 EtOAc–hexane). ^1H NMR (500 MHz, DMSO-d_6) δ 9.69 (br s, 2H, **H13**), 9.12 (br s, 2H, **H11**), 8.85 (s, 1H, **H9**), 8.66 (s, 1H, **H10**), 8.13 (d, *J* = 1.6 Hz, 4H, **H15**), 7.94 (d, *J* = 8.5 Hz, 2H, **H4** & **H5**), 7.76 (d, *J* = 6.7 Hz, 2H, **H2** & **H7**), 7.61 (d, *J* = 1.6 Hz, 2H, **H17**), 7.54 (dd, *J* = 8.5, 7.2 Hz, 2H, **H3** & **H6**). ^{13}C NMR (126 MHz, DMSO-d_6) δ 153.3 (**C12**), 141.9 (**C14**), 133.5 (**C1** & **C8**), 131.8 (**C4a** & **C5a**), 130.7 (q, *J* = 32.8 Hz, **C16**), 127.1 (**C10**), 126.3 (**C1a** & **C8a**), 125.6 (**C3** & **C6**), 124.8 (**C4** & **C5**), 123.3 (q, *J* = 272.6 Hz, **C18**), 119.9 (**C2** & **C7**), 117.8 (**C15**), 115.5 (**C9**), 114.3 (**C17**). ^{19}F NMR (377 MHz, DMSO-d_6) δ -61.8 (18-$\mathbf{CF_3}$). IR (neat) *v*/cm^{-1} 3353 (br w, N–H), 3301 (br w, N–H), 3094 (w, C–H), 2973 (w, C–H), 1738 (br m), 1655 (m), 1585 (m), 1564 (m), 1471 (m), 1382 (m), 1273 (s), 1131 (s). HRMS (ESI) *m/z* calcd for $C_{32}H_{18}F_{12}N_4NaO_2$ $[M+Na]^+$ 741.1130, found 741.1126.

*1,1'-(anthracene-1,8-diyl)bis(3-(3,5-bis(trifluoromethyl)phenyl)thiourea) (**7SF2**)*

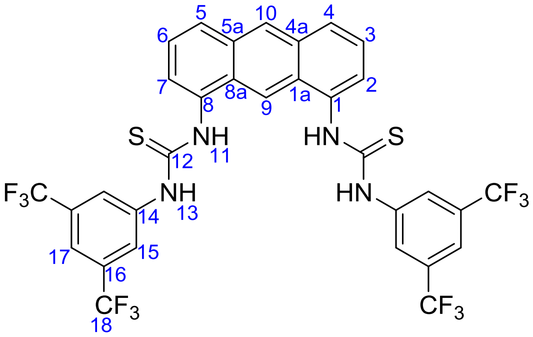

To a solution of 1,8-diaminoanthracene (**10**, 20 mg, 0.096 mmol) in anhydrous pyridine (1 mL) was added 3,5-bis(trifluoromethyl)phenyl isothiocyanate (53 µL, 0.288 mmol) and the resultant dark brown solution left to stir under N_2 at r.t. for 21 h. The solvent was removed *in vacuo* by co-evaporating with toluene (x3) to give a brown solid. This was purified by flash column chromatography (SiO_2; 1% MeOH in DCM) to give the title compound as a beige solid (44 mg, 61%). R_f 0.50 (EtOAc-hexane 1:1). ^{1}H NMR (500 MHz, acetone-d_6) δ 9.83 (br s, 2H, **H11**), 9.52 (br s, 2H, **H13**), 9.11 (s, 1H, **H9**), 8.73 (s, 1H, **H10**), 8.30 (s, 4H, **H15**), 8.13 (d, *J* = 8.5 Hz, 2H, **H4** & **H5**), 7.70 (d, *J* = 7.0 Hz, 4H, **H2** & **H7** [overlaps with **H17**]), 7.60 (dd, *J* = 8.6, 7.1 Hz, 2H, **H3** & **H6**). ^{13}C NMR (126 MHz, acetone-d_6) δ 183.0 (**C12**), 142.7 (**C14**), 135.0 (**C1** & **C8**), 133.8 (**C4a** & **C5a**), 131.6 (q, *J* = 33.2 Hz, **C16**), 129.8 (**C1a** & **C8a**), 129.1 (**C4** & **C5**), 128.4 (**C10**), 126.6 (**C3** & **C6**), 126.5 (**C2** & **C7**), 125.7 (**C15**), 124.3 (q, *J* = 272.0 Hz, **C18**), 118.5 (**C9** & **C17**). ^{19}F NMR (377 MHz, acetone-d_6) δ -63.4 (18-$\mathbf{CF_3}$). IR (neat) v/cm^{-1} 3375 (w, N–H), 3146 (br w, N–H), 2973 (w, C–H), 2933 (w, C–H), 1624 (w), 1539 (m), 1383 (br m), 1276 (m), 1125 (s). HRMS (ESI) *m/z* calcd for $C_{32}H_{18}F_{12}N_4NaS_2$ [M+Na]$^+$ 773.0673, found 773.0679.

2.3. NMR Spectra

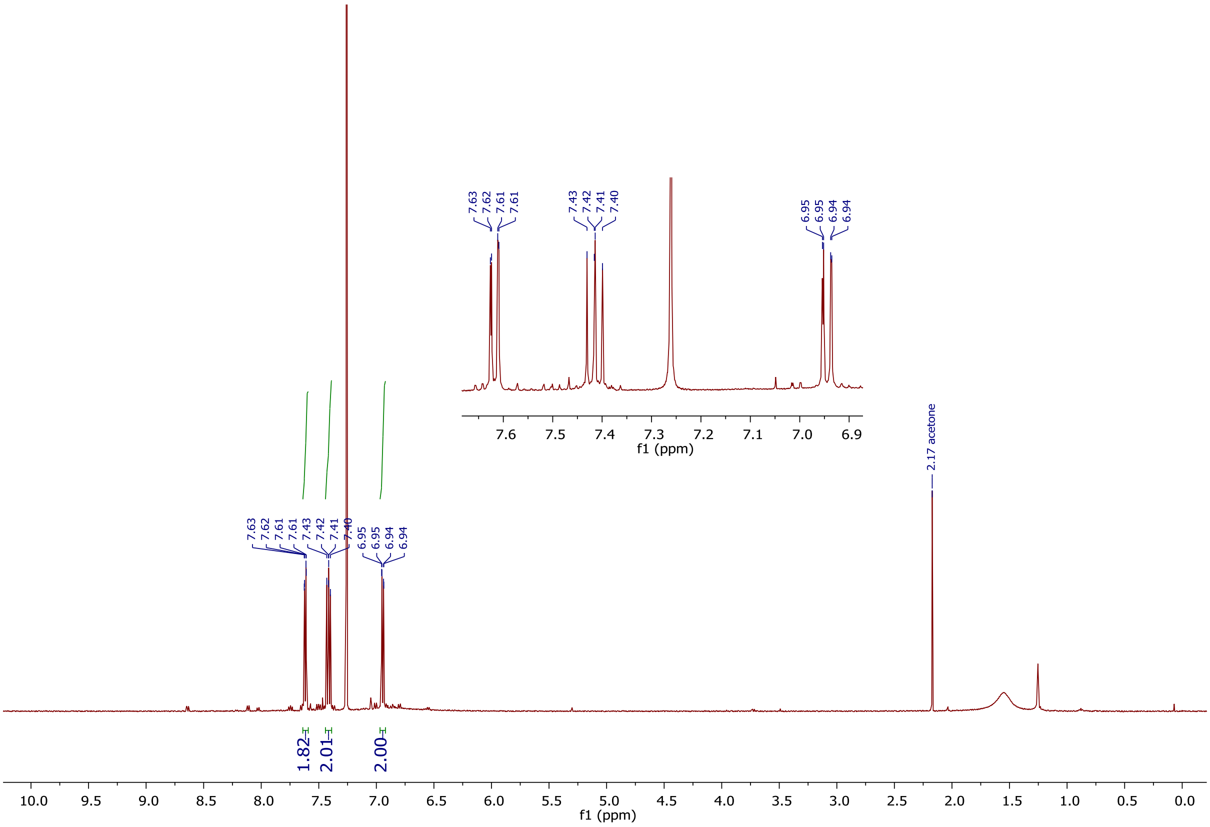

Figure S2. ^{1}H NMR spectrum of **9** in $CDCl_3$.

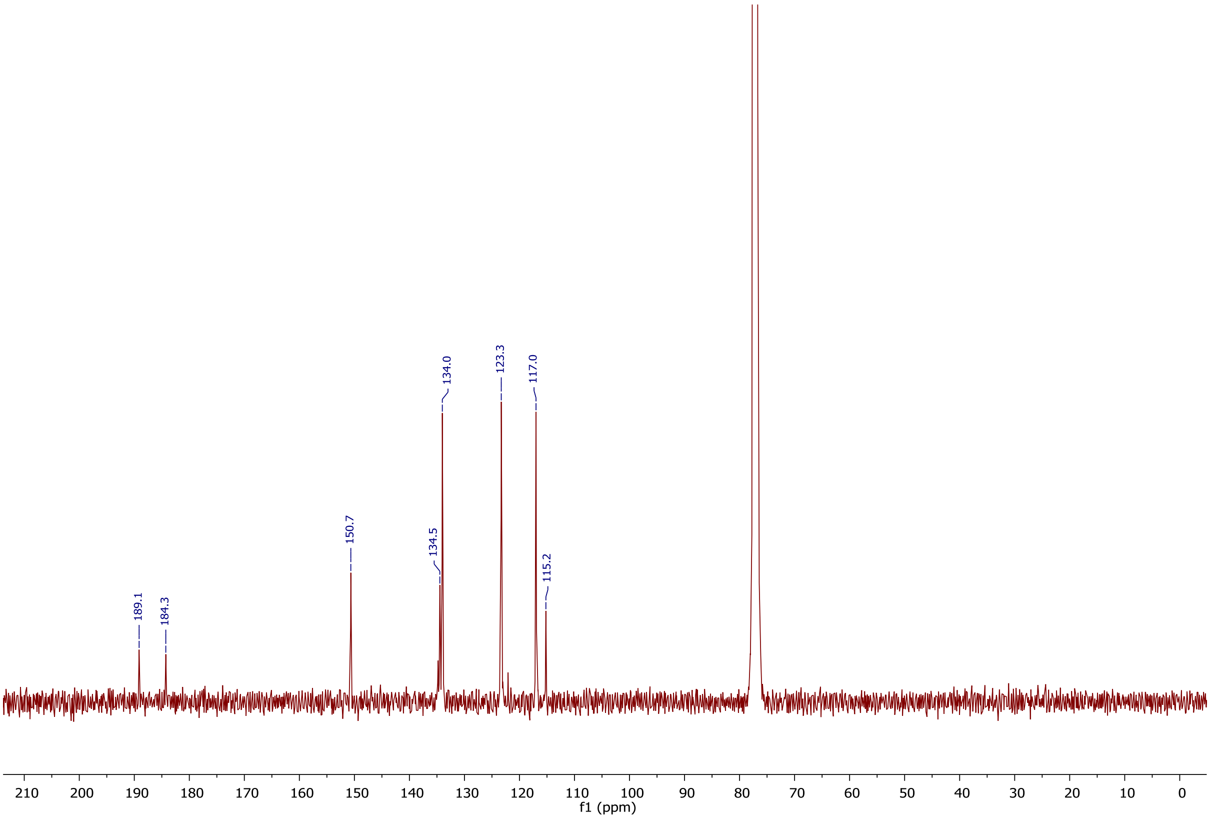

Figure S3. ^{13}C NMR spectrum of **9** in $CDCl_3$.

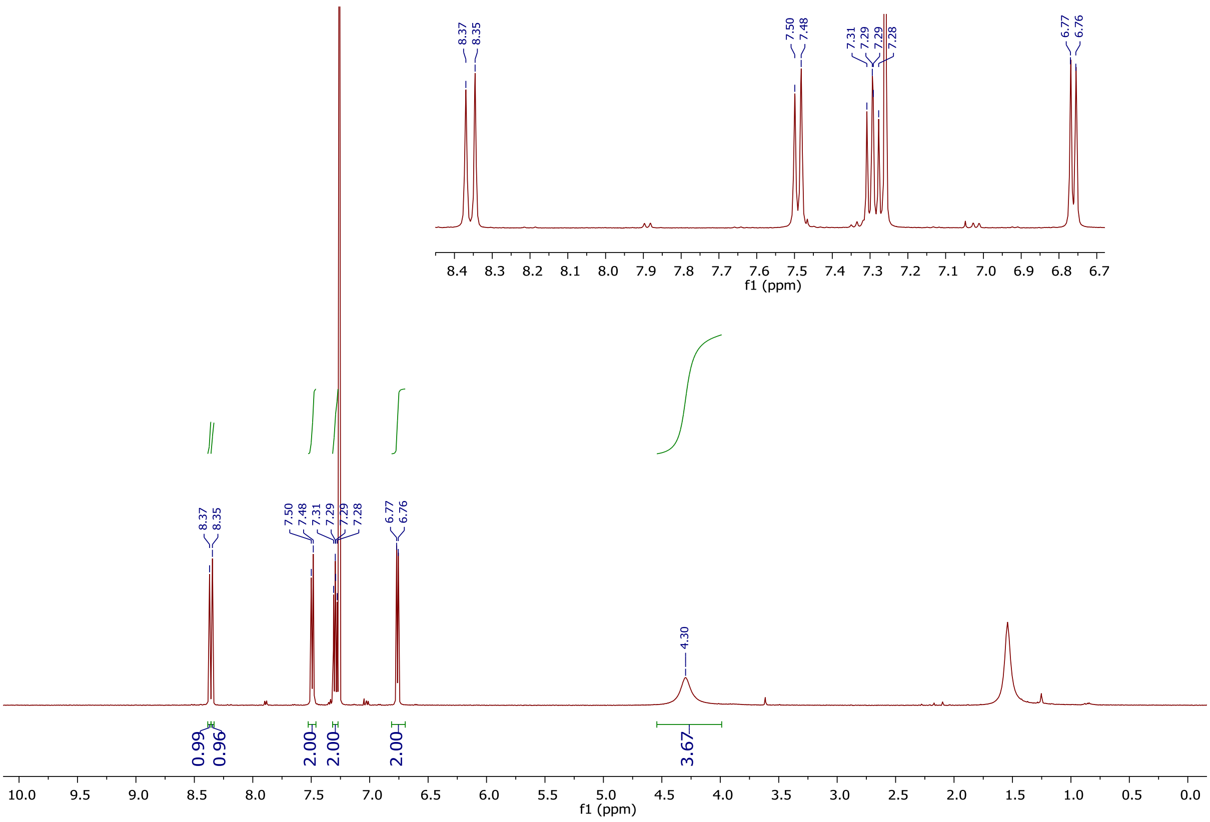

Figure S4. ^{1}H NMR spectrum of **10** in $CDCl_3$.

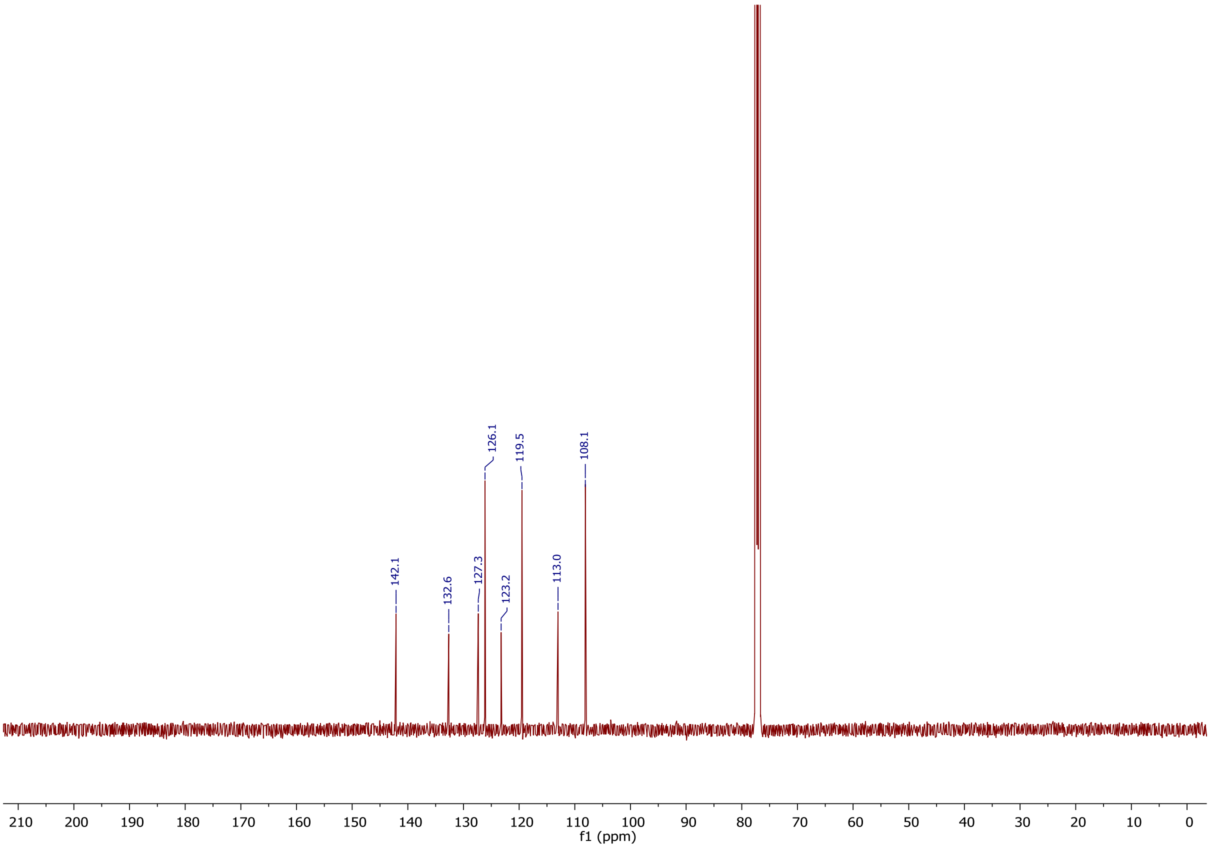

Figure S5. ^{13}C NMR spectrum of **10** in $CDCl_3$.

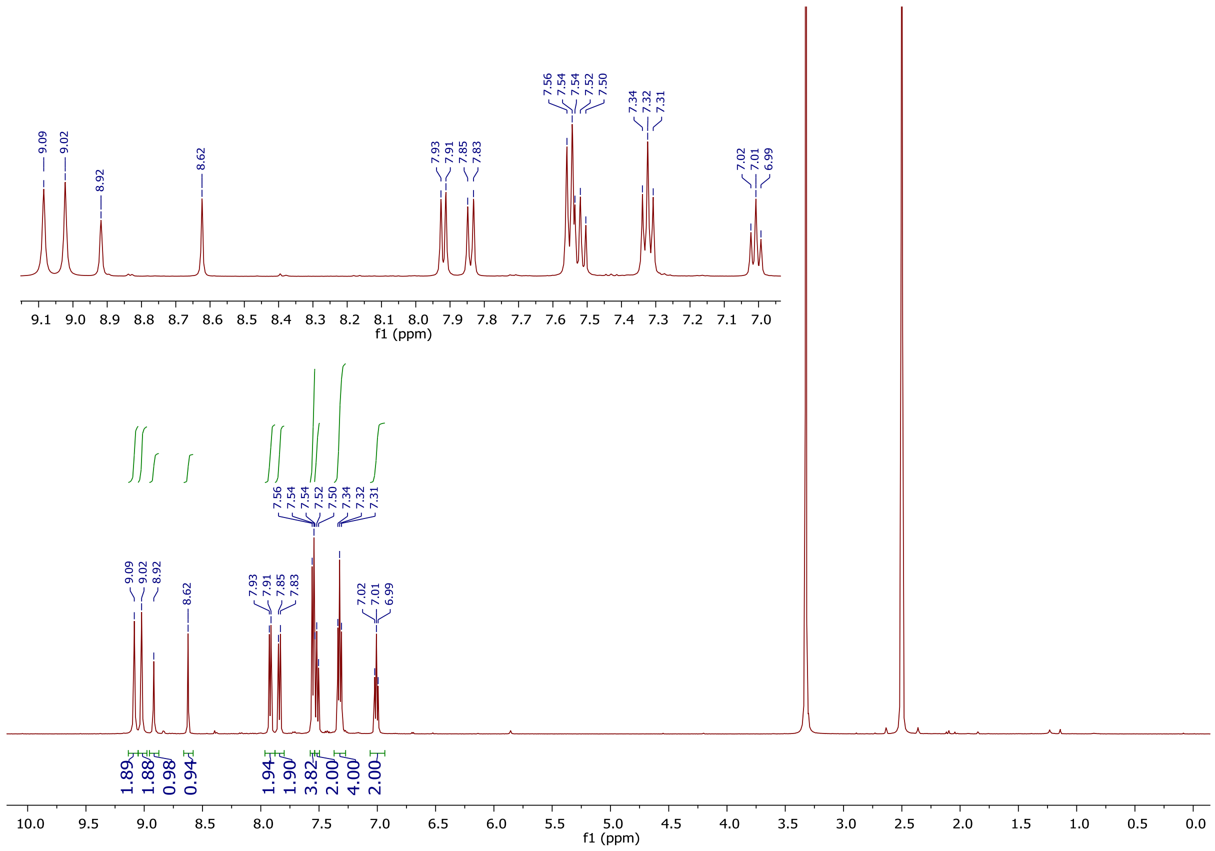

Figure S6. ^{1}H NMR spectrum of **7OP** in DMSO-d_6.

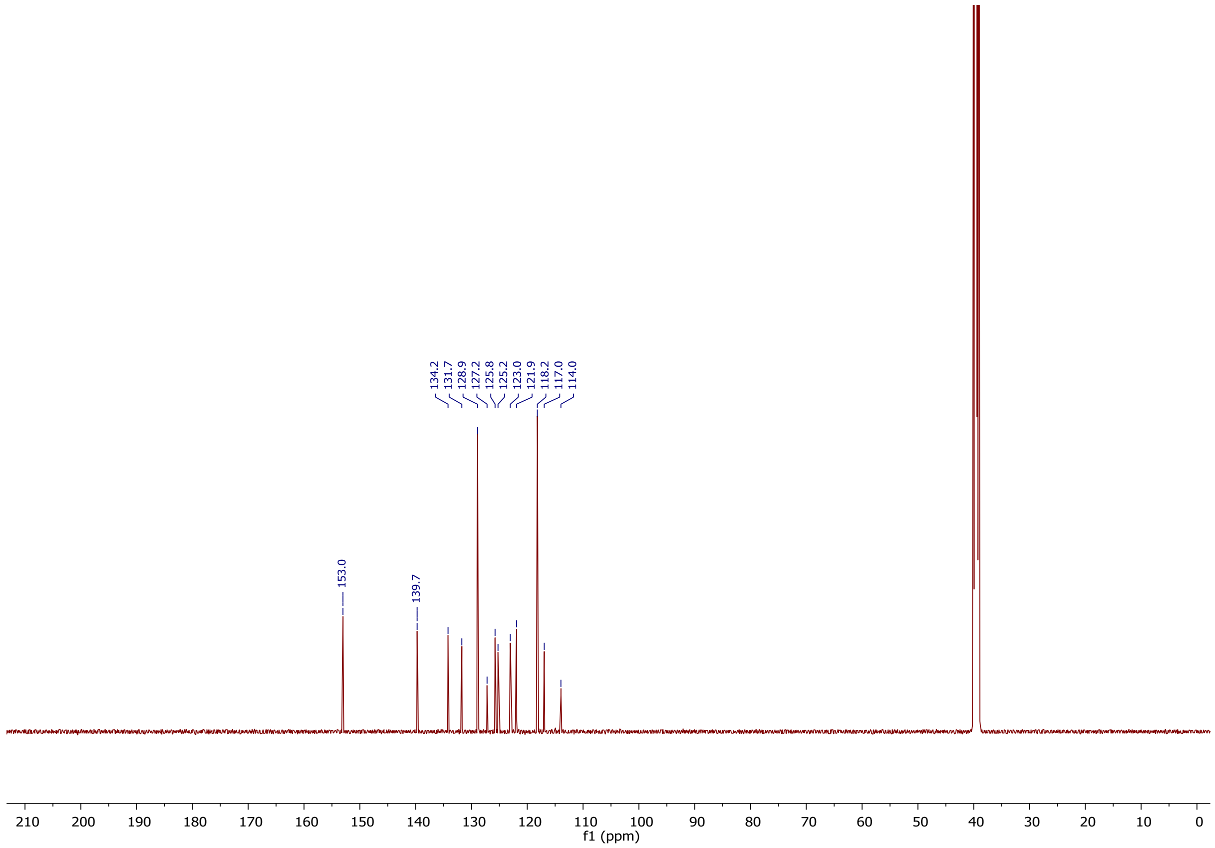

Figure S7. ^{13}C NMR spectrum of **7OP** in DMSO-d_6.

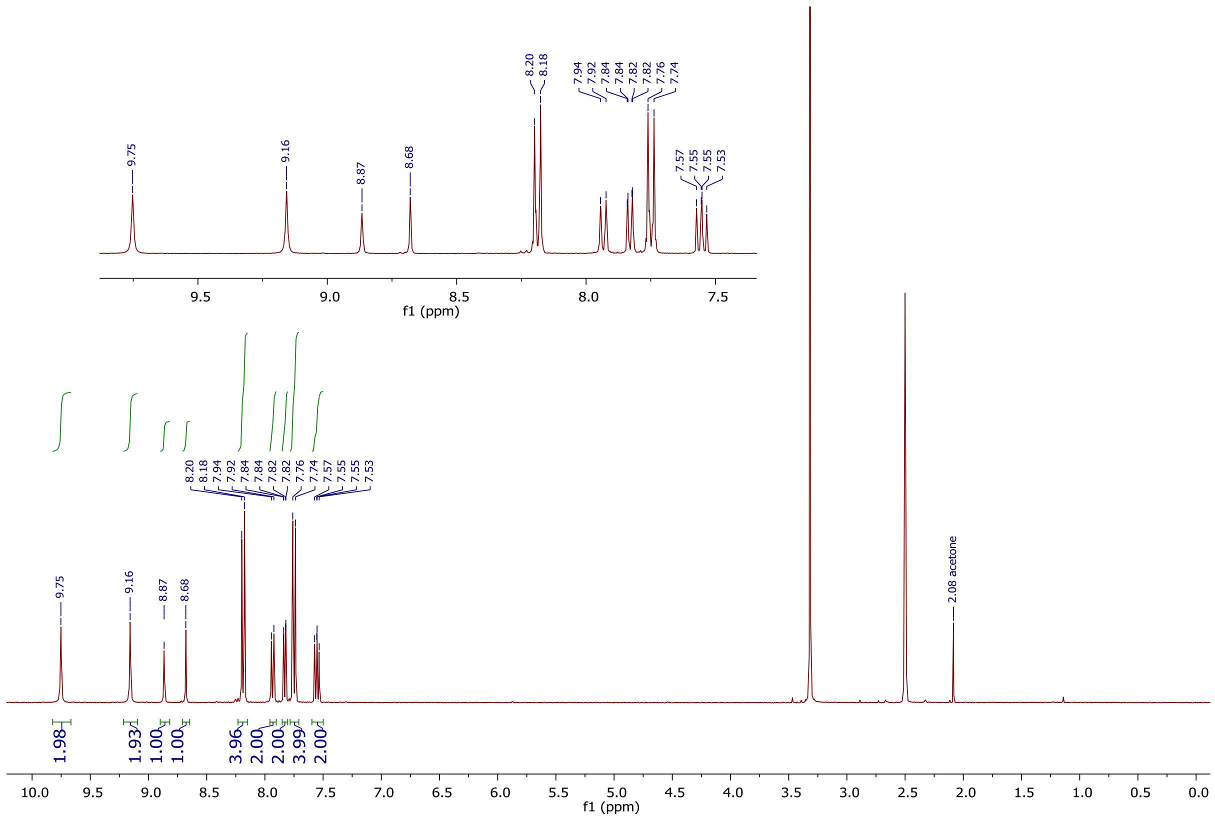

Figure S8. ^{1}H NMR spectrum of **7ON** in DMSO-d_6.

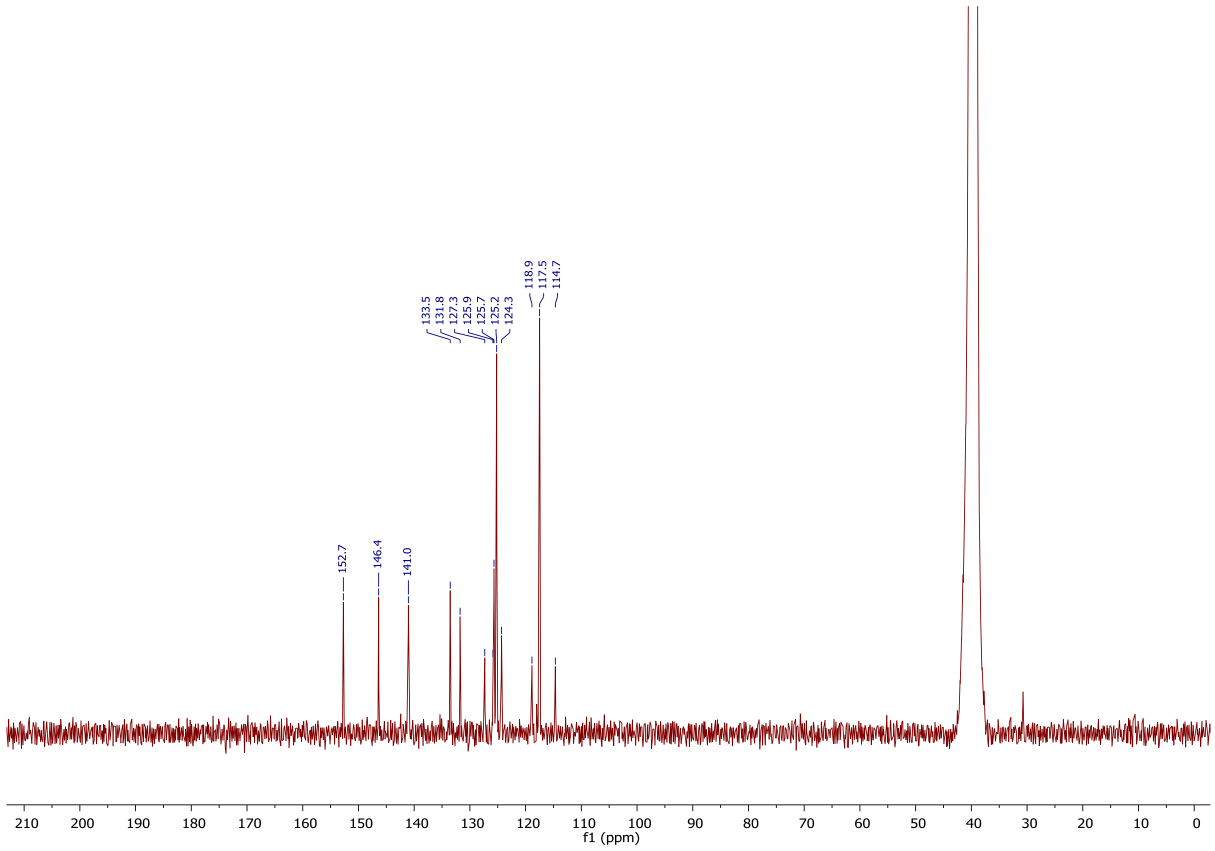

Figure S9. ^{13}C NMR spectrum of **7ON** in DMSO-d_6.

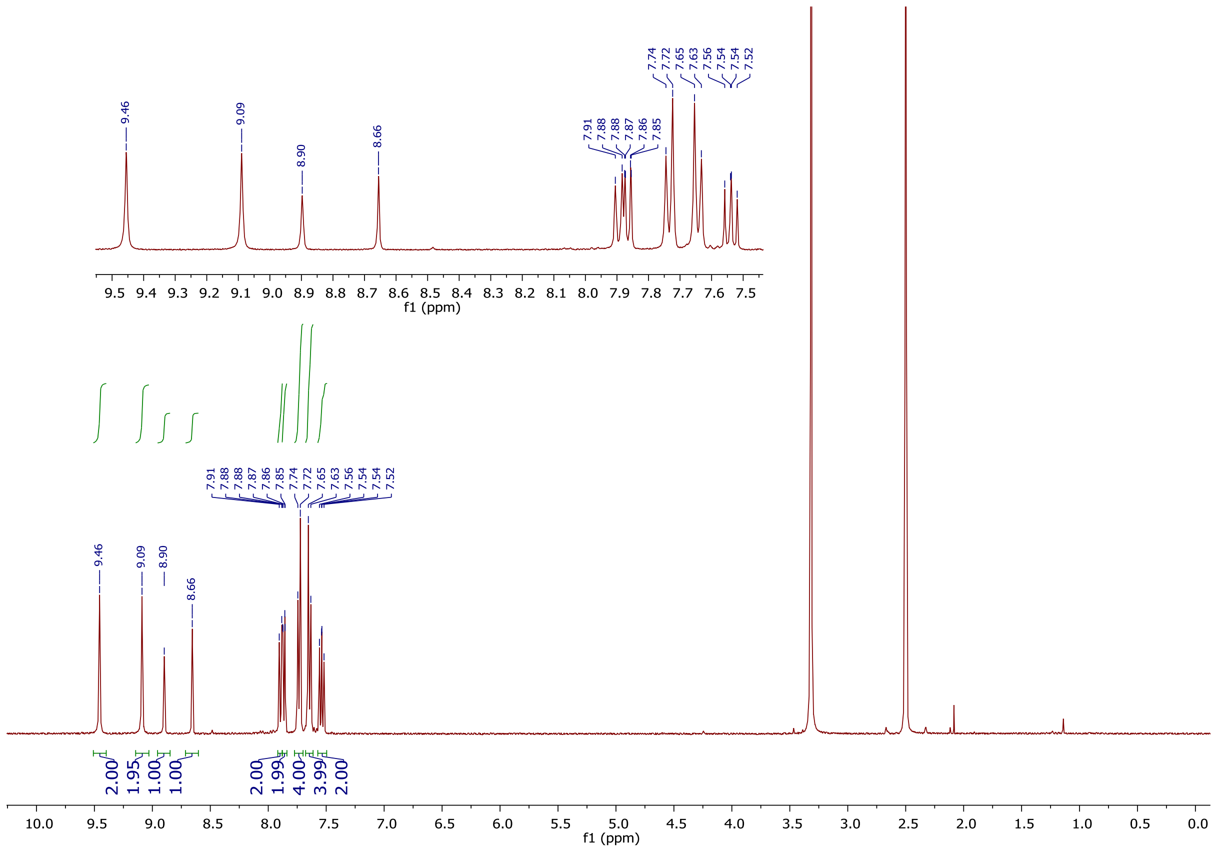

Figure S10. ^{1}H NMR spectrum of **7OF** in DMSO-d_6.

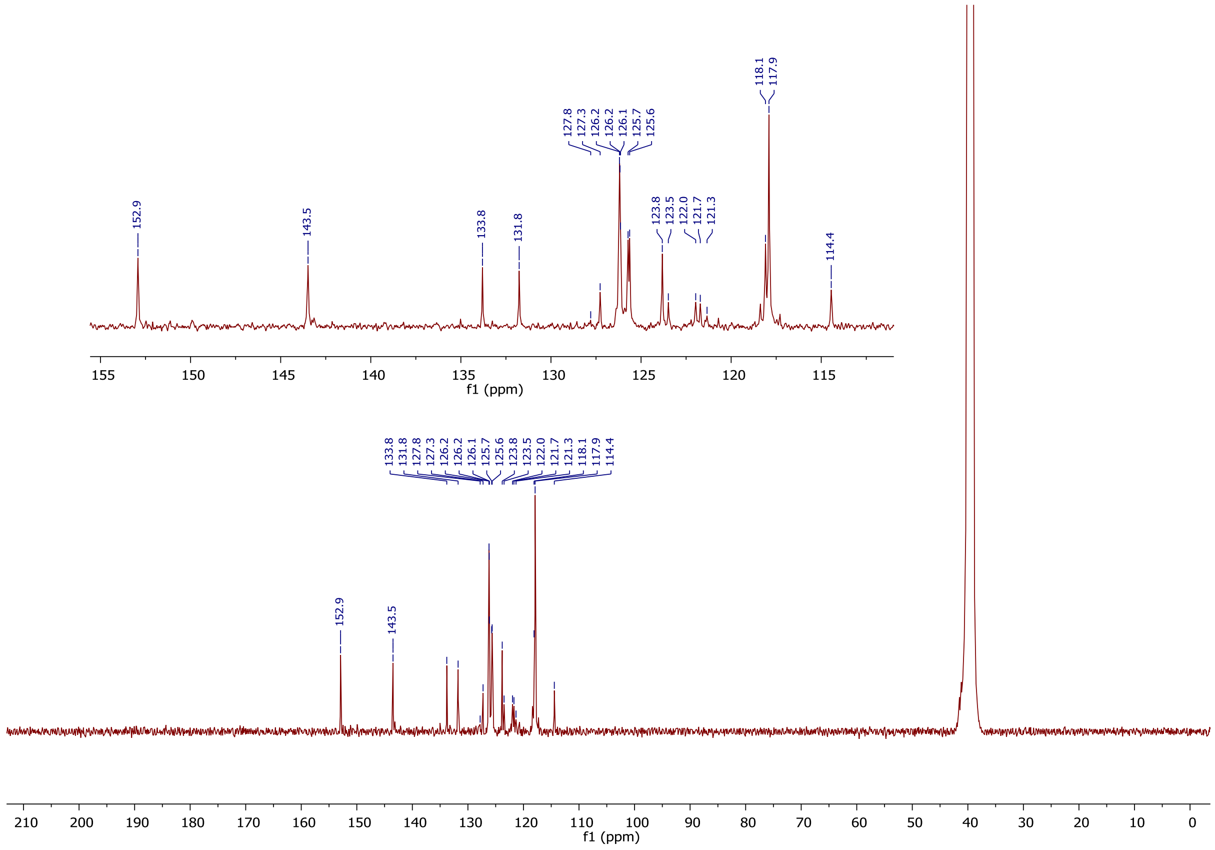

Figure S11. ^{13}C NMR spectrum of **7OF** in DMSO-d_6.

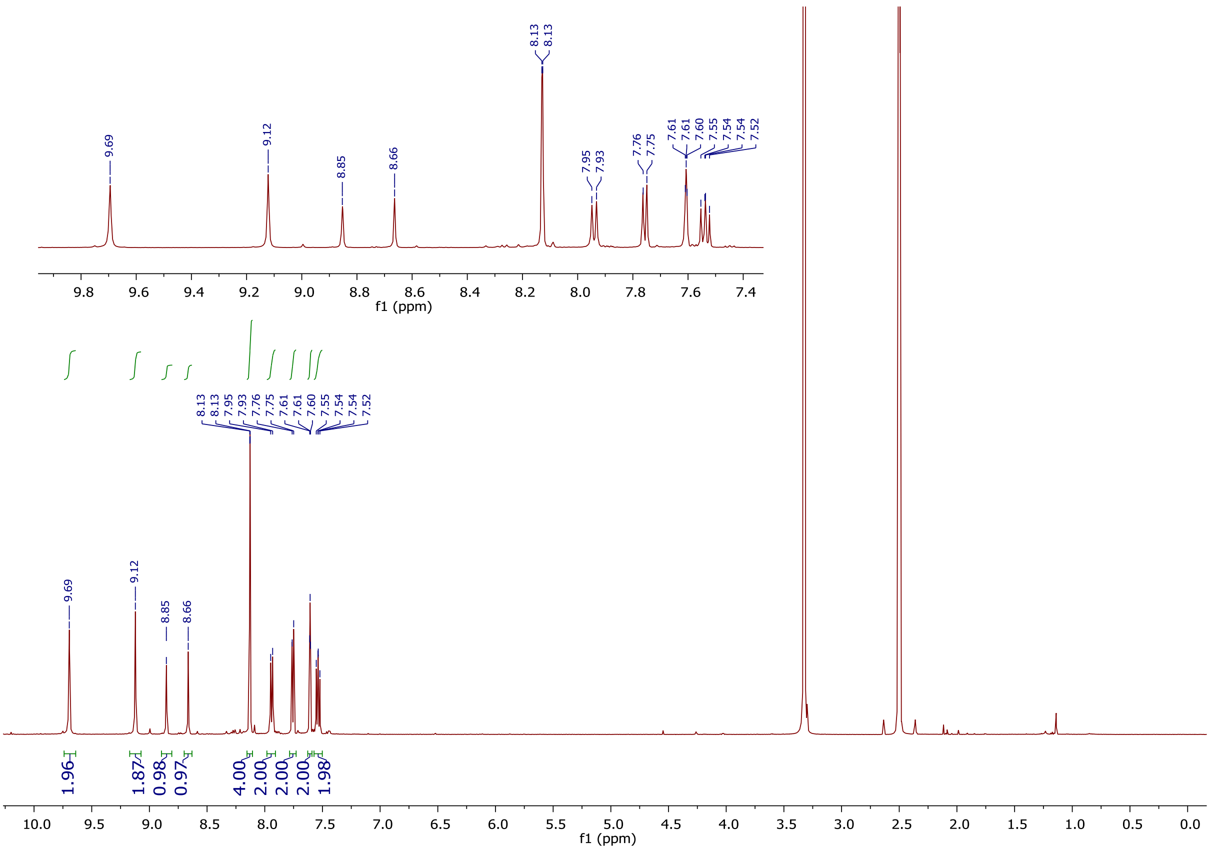

Figure S12. ^{1}H NMR spectrum of **7OF2** in DMSO-d_6.

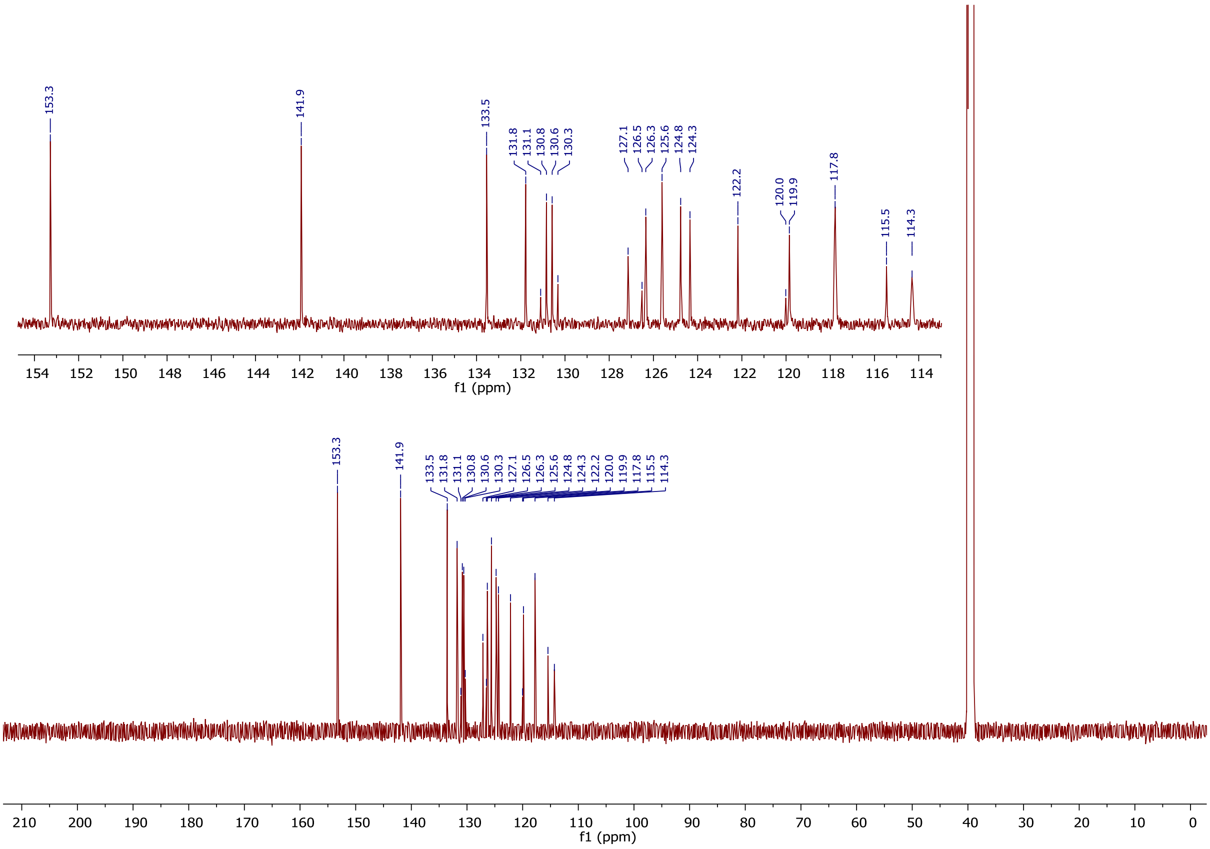

Figure S13. ^{13}C NMR spectrum of **7OF2** in DMSO-d_6.

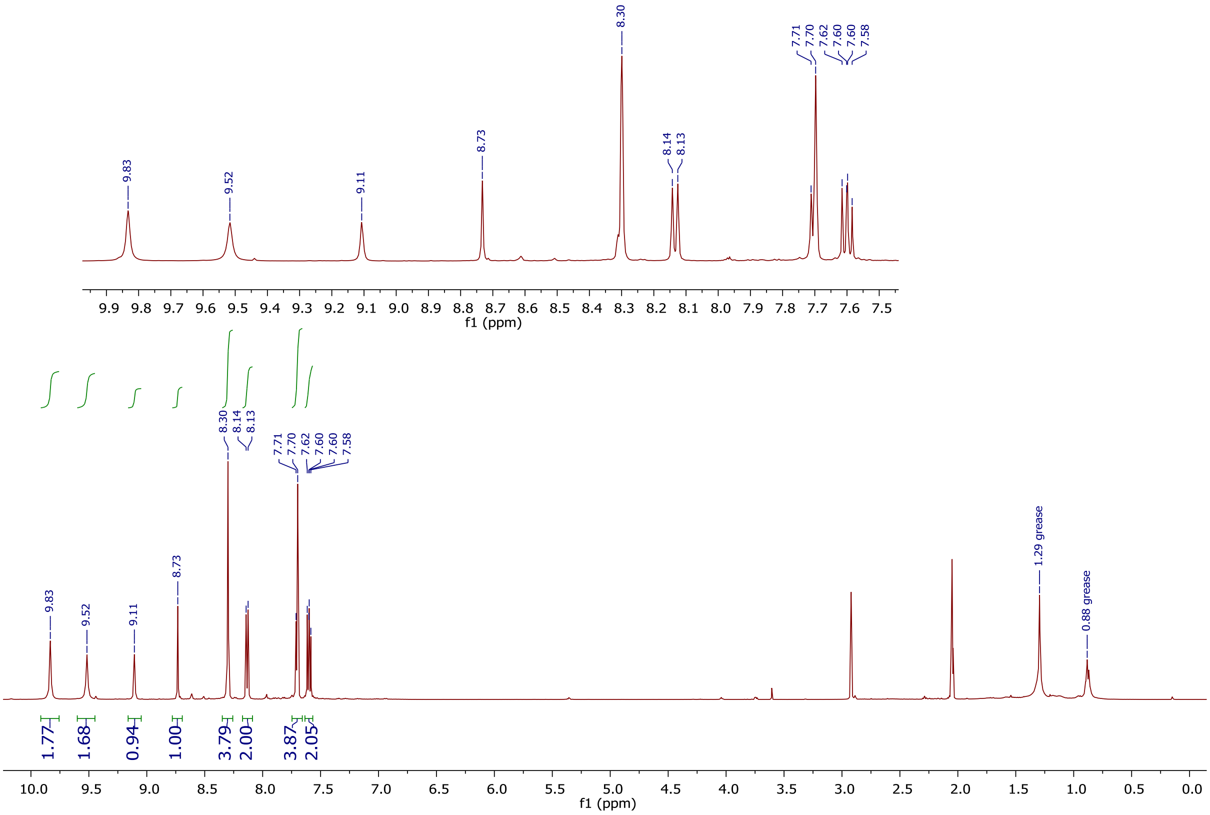

Figure S14. ^{1}H NMR spectrum of **7SF2** in acetone-d_6.

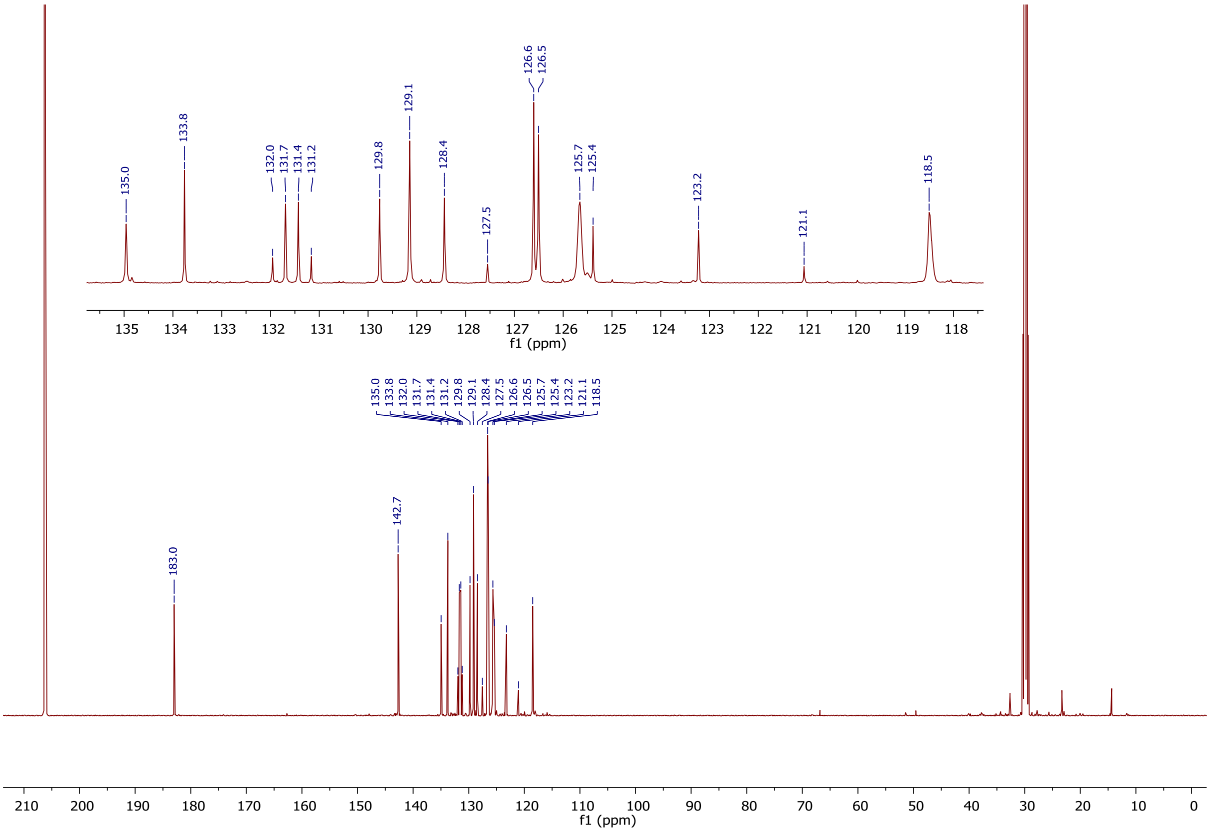

Figure S15. ^{13}C NMR spectrum of **7SF2** in acetone-d_6.

3. Determination of Chloride Binding Affinities through ^{1}H NMR Titrations

3.1. Method

Binding constants were measured by titrating n-$Bu_4N^+Cl^-$ into a solution of the host in DMSO-d_6/0.5% H_2O at 298 K. A stock solution of the host (~1 mM) in Millipore water (10 µL) and DMSO-d_6 (1.99 mL) was prepared. A stock solution of the guest was prepared by dissolving n-$Bu_4N^+Cl^-$ (previously dried under high vacuum) in the aforementioned stock solution of host (1 mL). The concentration of the guest stock solution was varied over different experiments. The host solution (500 µL) was transferred to an NMR tube and an initial NMR spectrum recorded (Varian VNMRS 500b (proton sensitive) spectrometer). Thereafter, aliquots of the guest solution were added to the NMR tube and a spectrum recorded after each addition.

As described in the main text, binding stoichiometries were determined by visually comparing the residual distribution plots for each receptor when titration data were fitted to 1:1, 1:2 and 2:1 binding models (see Figures S18, S21, S24, S27 and S30). A stochastic distribution of residuals with very low values was observed when data were fit to a 2:1 (host:guest) model for bisureas **7OP**, **7ON**, **7OF** and **7OF2** and a 1:2 model in the case of bisthiourea **7SF2**. In contrast, a regular, sinusoidal distribution of residuals was observed when the data were fit to either 1:1 or 1:2 models in the case of the bisureas or 1:1 and 2:1 models in the case of **7SF2**. These results suggest that the most likely mode of binding is a 2:1 stoichiometry for **7OP**, **7ON**, **7OF** and **7OF2** and a 1:2 stoichiometry for **7SF2**.[5,6,7]

Consequently, the shift of both N*H* signals were fit to either a 2:1 binding model (Nelder-Mead method) in the case of the bisureas or a 1:2 model in the case of **7SF2** using the Bindfit v0.5 applet (available as freeware from Supramolecular.org).[†] The binding constants obtained are reported in the main text (Table 1) and the fits to the most downfield N*H* signal are shown in Figures S17, S20, S23, S26 and S29.

[†] Accessed on 8 January 2018.

3.2. NMR Stackplots, Binding Curves and Residual Distribution Plots

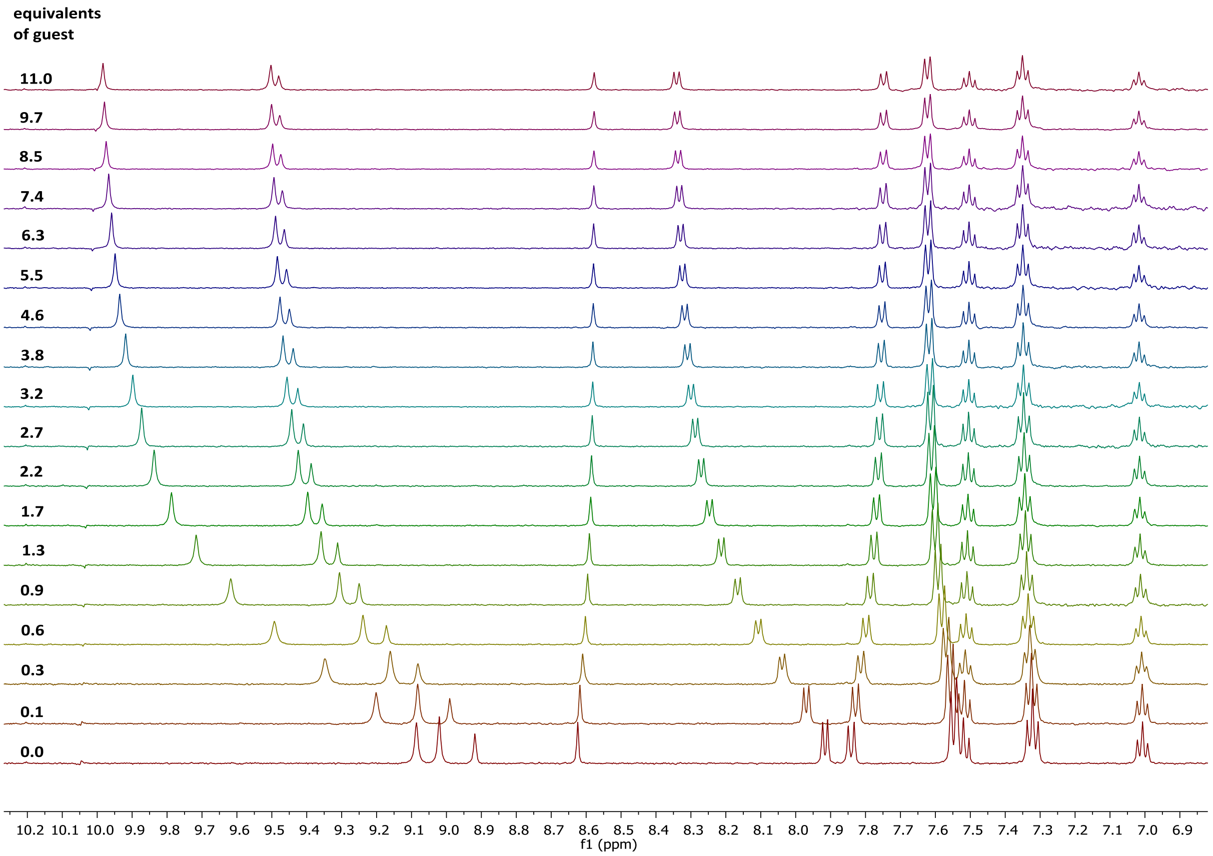

Figure S16. Downfield region of the ^{1}H NMR spectra for the titration of $Bu_4N^{+}Cl^{-}$ into **7OP** in DMSO-d_6/0.5% H_2O at 298 K.

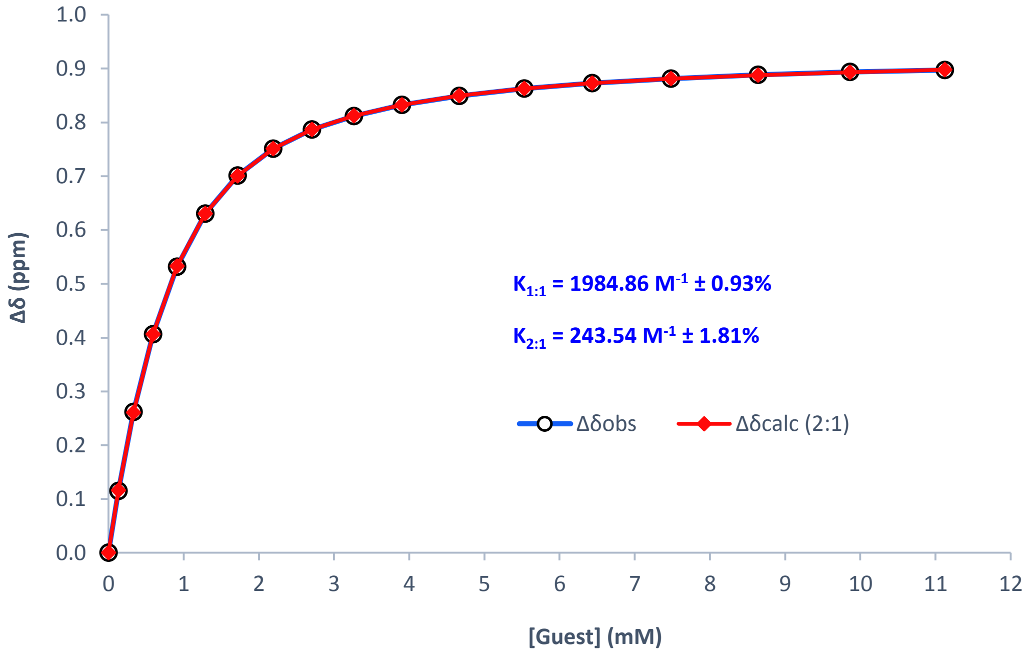

Figure S17. Observed binding curve (N*H*; starting at 9.09 ppm) and calculated fit (2:1 binding model) for **7OP** when titrated against $Bu_4N^{+}Cl^{-}$ in DMSO-d_6/0.5% H_2O at 298 K.

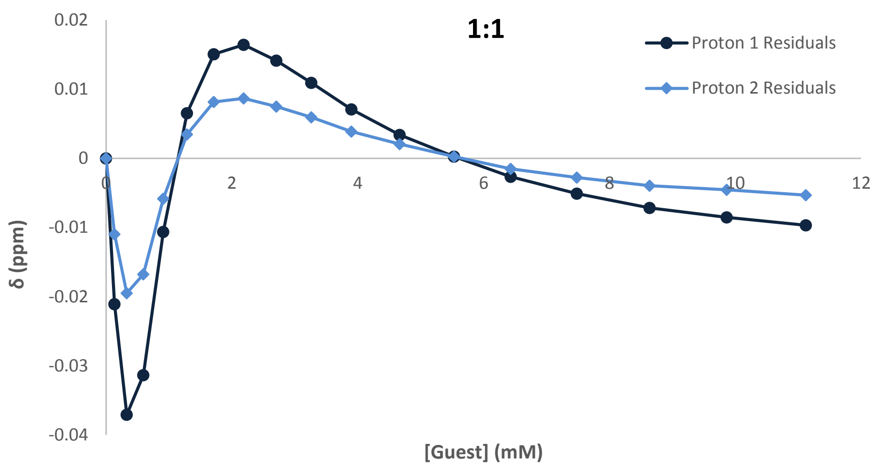

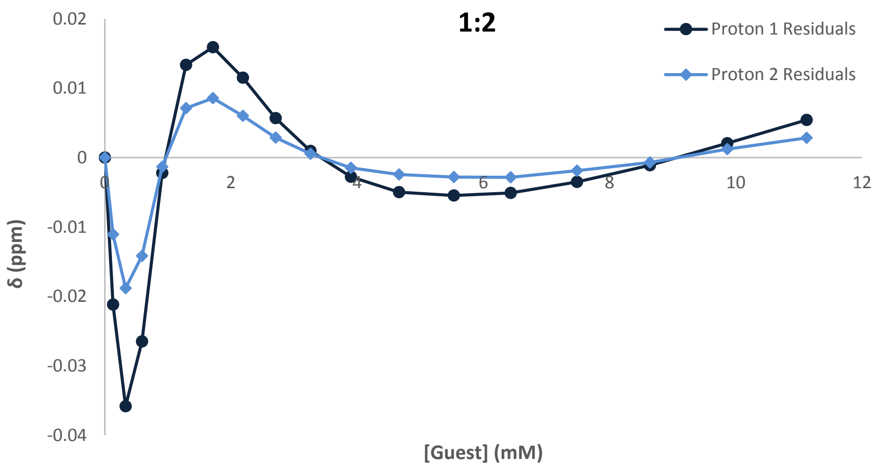

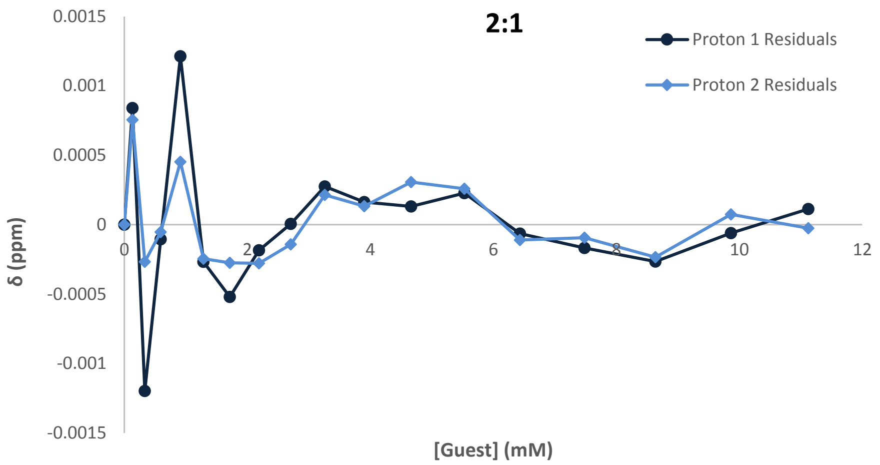

Figure S18. Residual distribution plots for **7OP** when titration data were fit to 1:1 (top), 1:2 (middle) and 2:1 (bottom) binding models. Proton 1 (N*H*; starting at 9.09 ppm) and proton 2 (N*H*; starting at 9.02 ppm) were monitored.

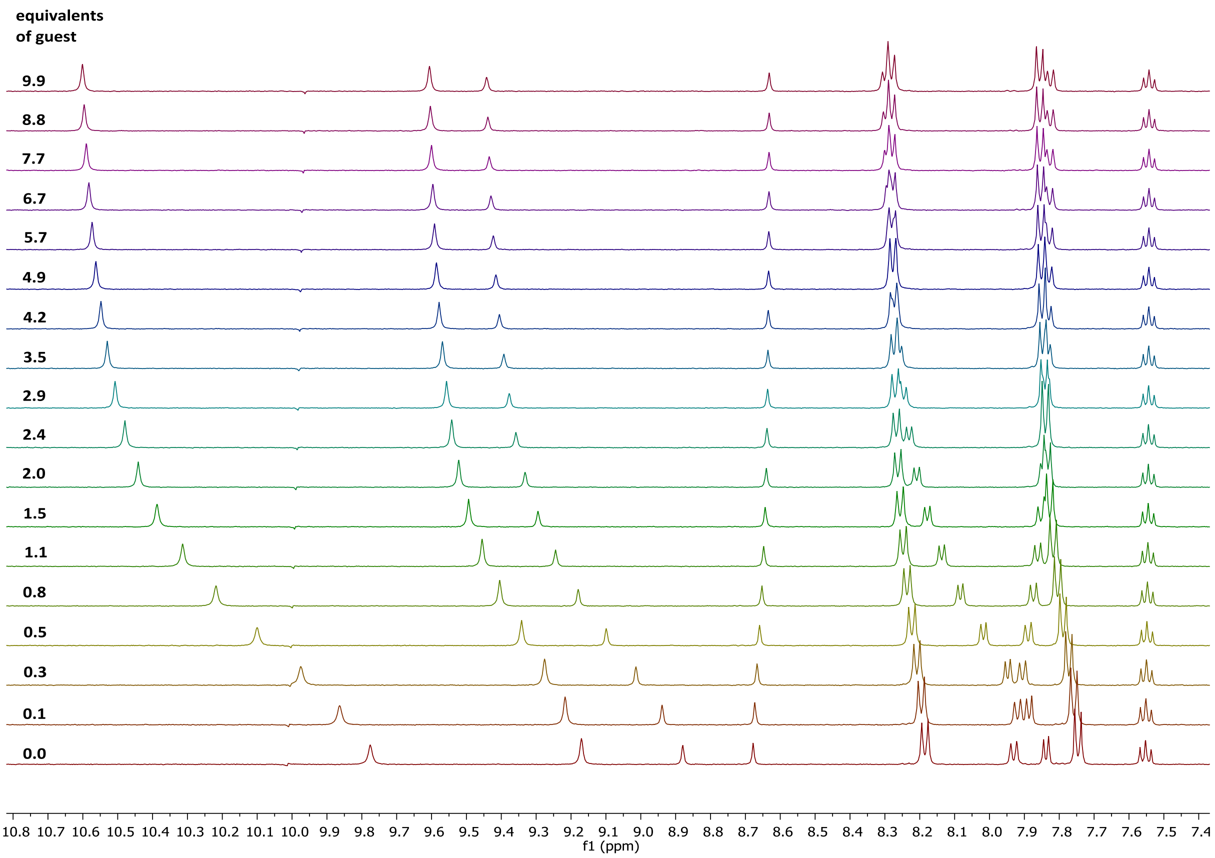

Figure S19. Downfield region of the ^{1}H NMR spectra for the titration of $Bu_4N^+Cl^-$ into **7ON** in DMSO-d_6/0.5% H_2O at 298 K.

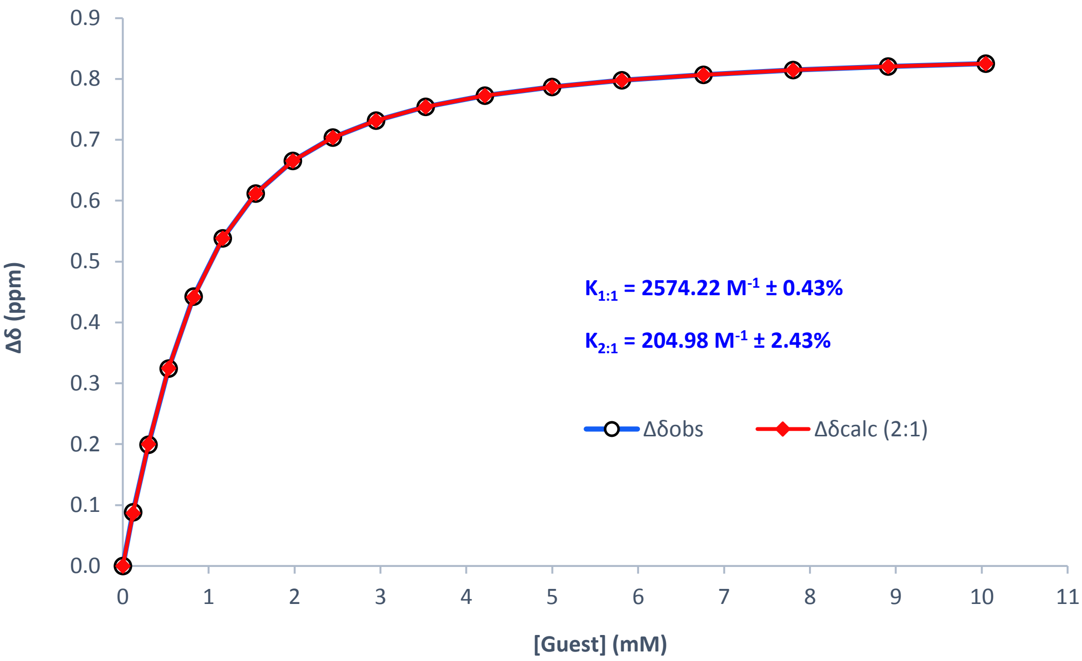

Figure S20. Observed binding curve (N*H*; starting at 9.78 ppm) and calculated fit (2:1 binding model) for **7ON** when titrated against $Bu_4N^+Cl^-$ in DMSO-d_6/0.5% H_2O at 298 K.

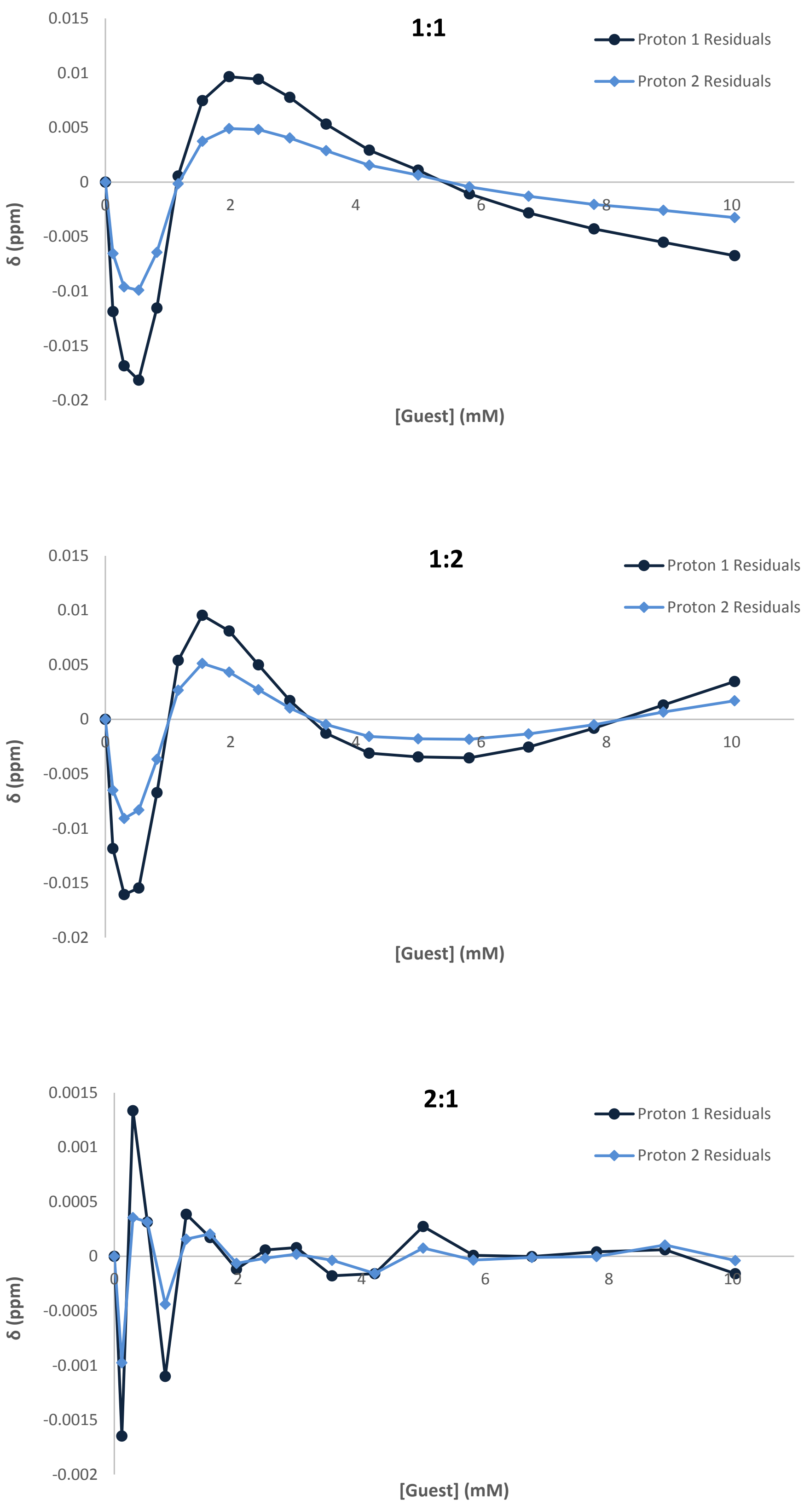

Figure S21. Residual distribution plots for **7ON** when titration data were fit to 1:1 (top), 1:2 (middle) and 2:1 (bottom) binding models. Proton 1 (N*H*; starting at 9.78 ppm) and proton 2 (N*H*; starting at 9.17 ppm) were monitored.

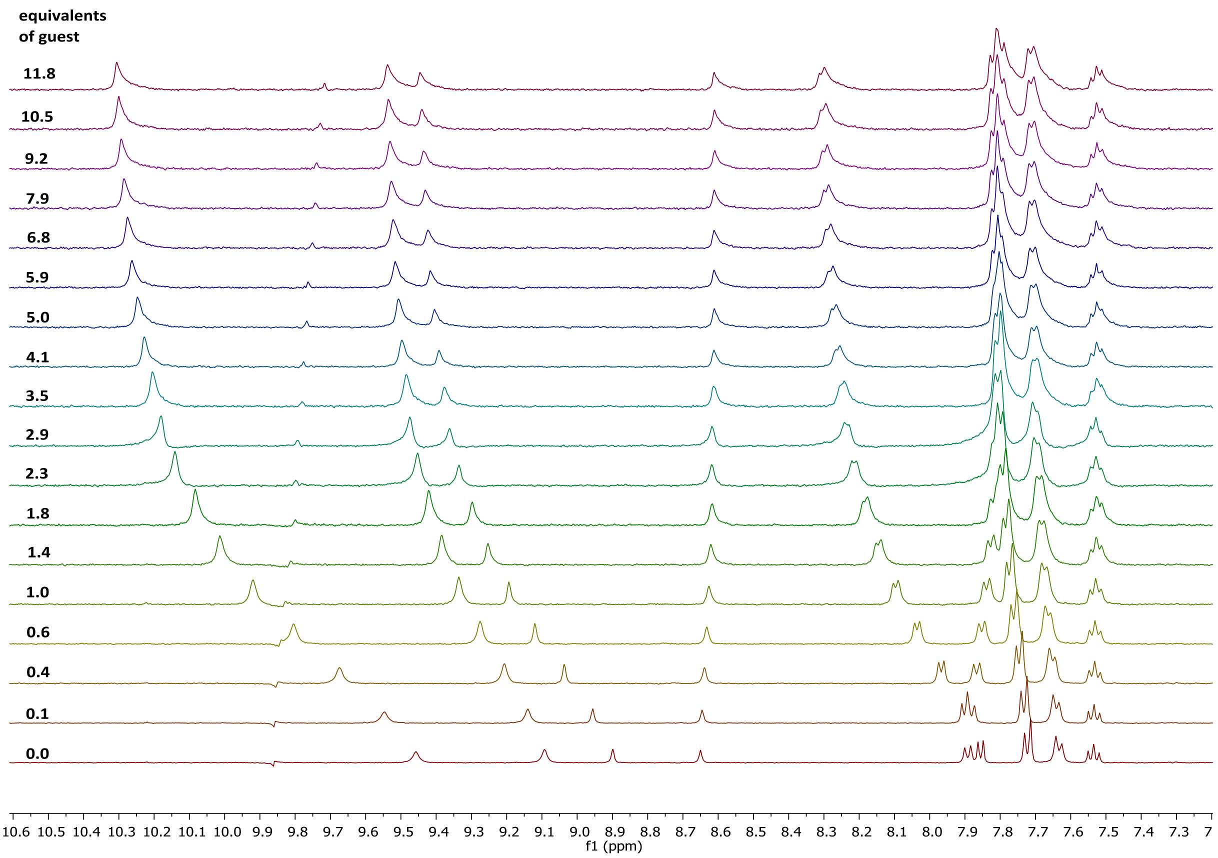

Figure S22. Downfield region of the ^{1}H NMR spectra for the titration of $Bu_4N^+Cl^-$ into **7OF** in DMSO-d_6/0.5% H_2O at 298 K.

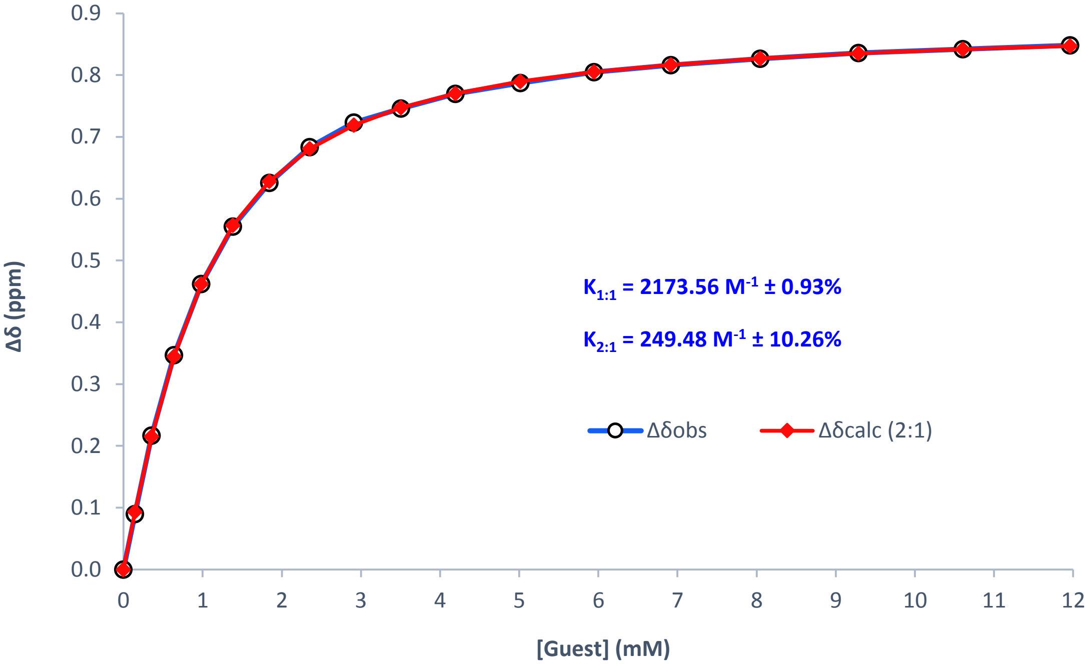

Figure S23. Observed binding curve (N*H*; starting at 9.46 ppm) and calculated fit (2:1 binding model) for **7OF** when titrated against $Bu_4N^+Cl^-$ in DMSO-d_6/0.5% H_2O at 298 K.

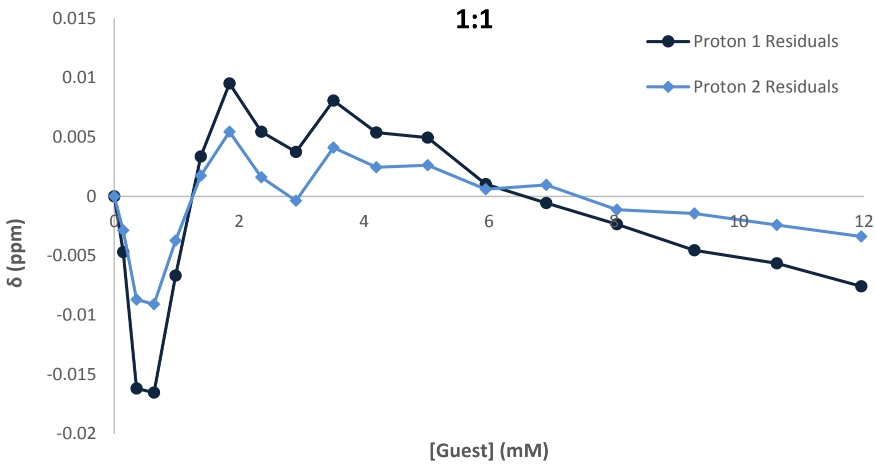

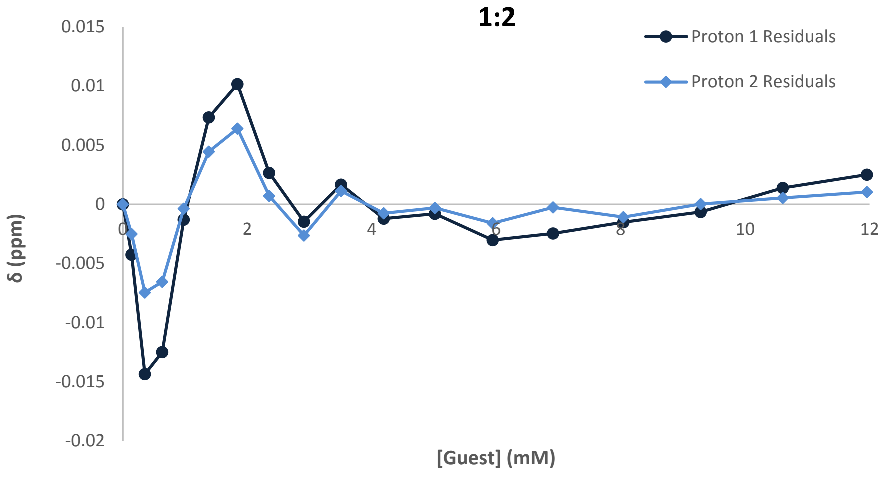

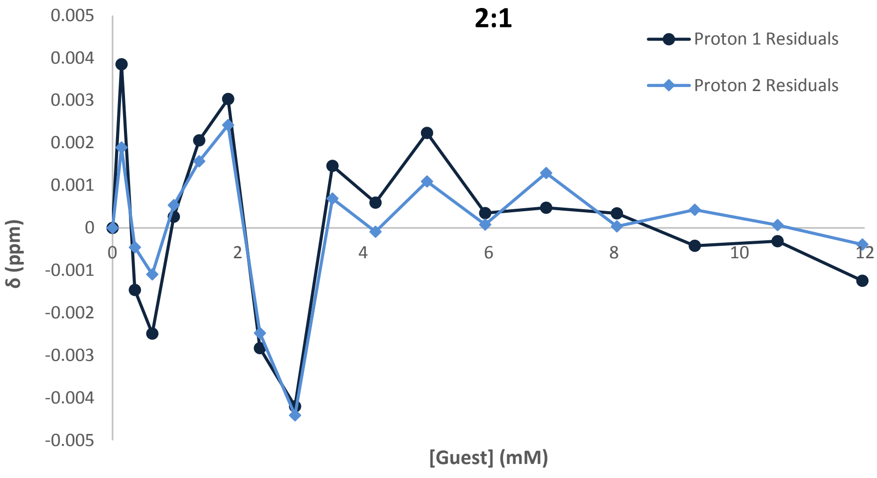

Figure S24. Residual distribution plots for **7OF** when titration data were fit to 1:1 (top), 1:2 (middle) and 2:1 (bottom) binding models. Proton 1 (N*H*; starting at 9.46 ppm) and proton 2 (N*H*; starting at 9.09 ppm) were monitored.

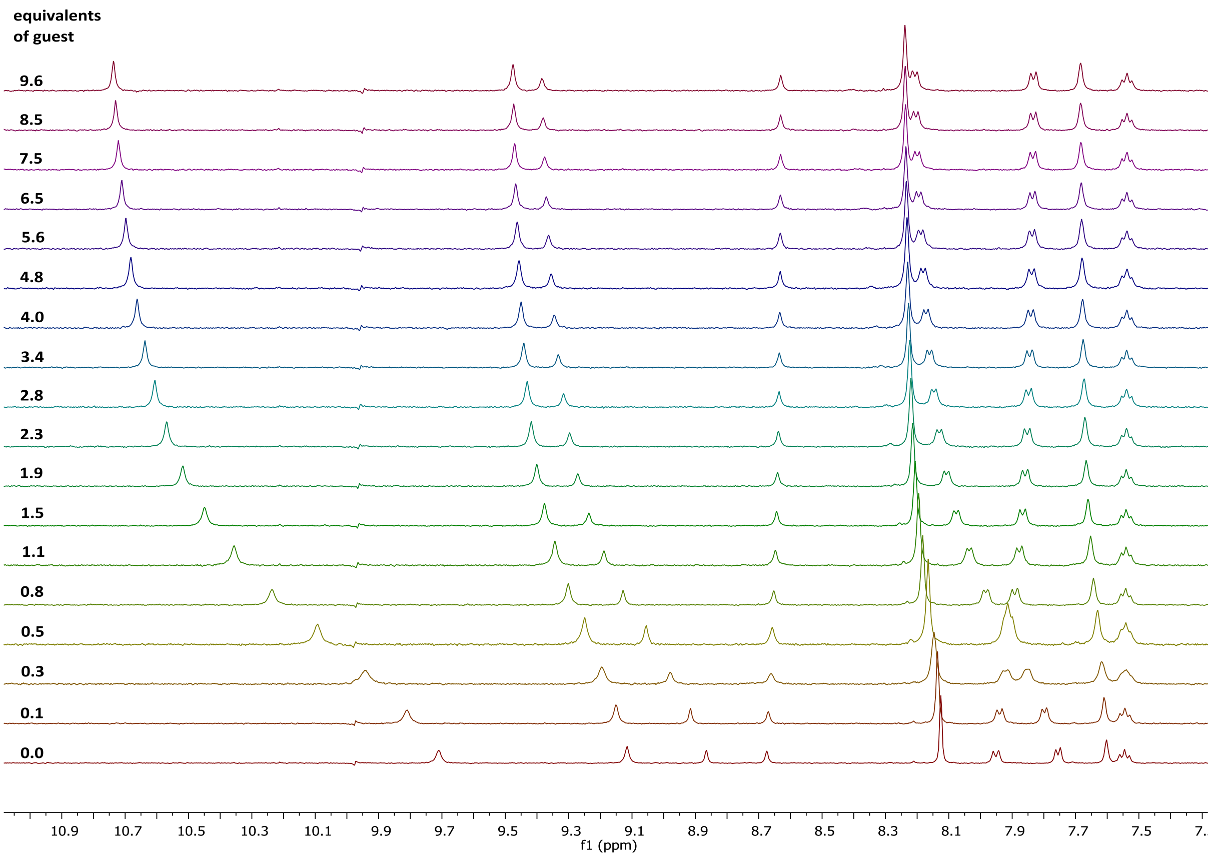

Figure S25. Downfield region of the ^{1}H NMR spectra for the titration of $Bu_4N^+Cl^-$ into **7OF2** in DMSO-d_6/0.5% H_2O at 298 K.

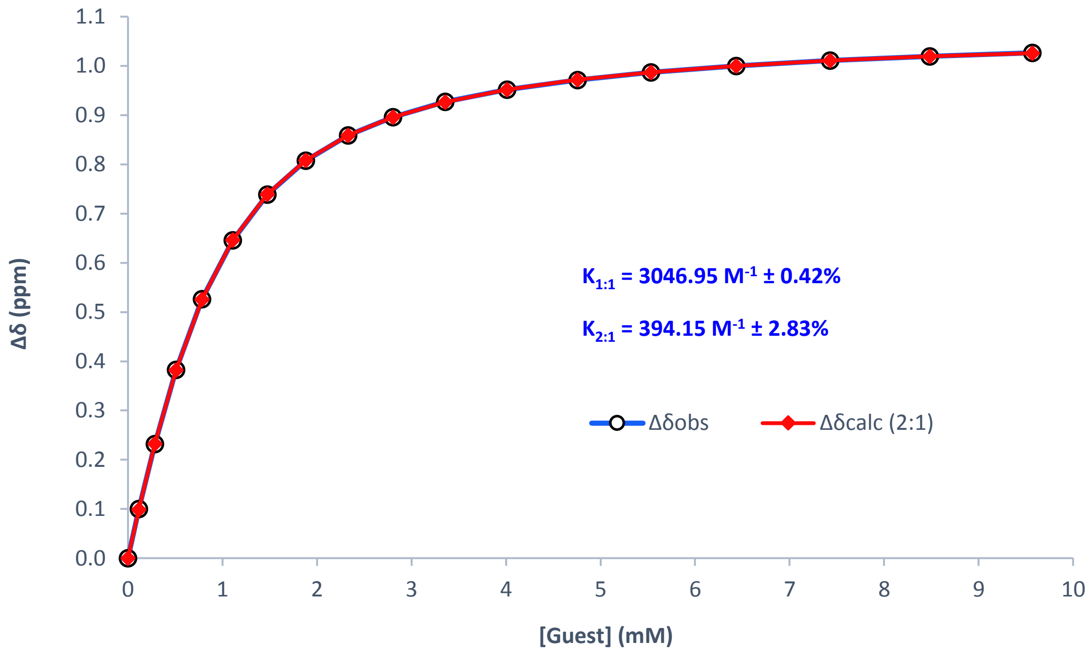

Figure S26. Observed binding curve (N*H*; starting at 9.71 ppm) and calculated fit (2:1 binding model) for **7OF2** when titrated against $Bu_4N^+Cl^-$ in DMSO-d_6/0.5% H_2O at 298 K.

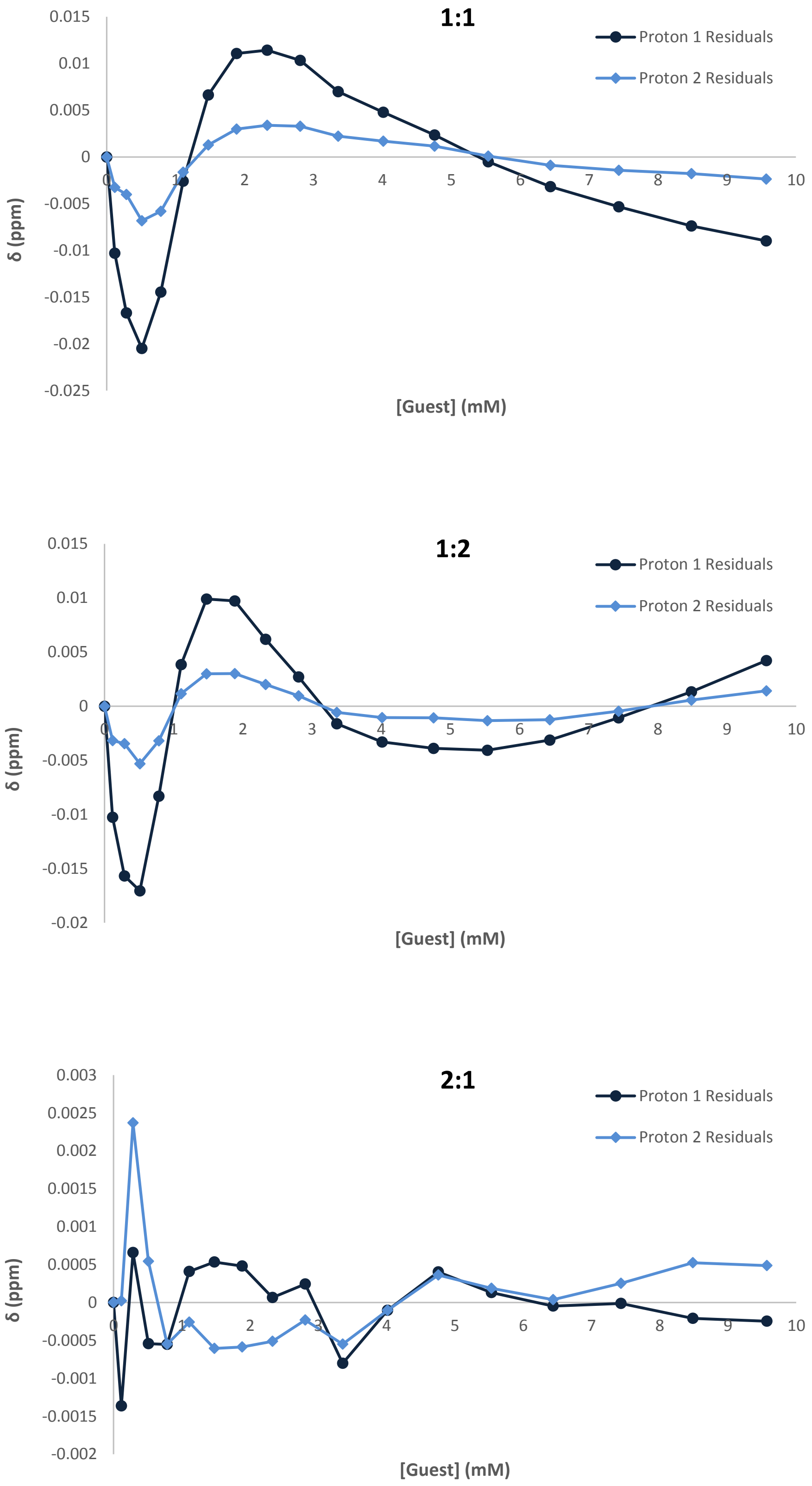

Figure S27. Residual distribution plots for **7OF2** when titration data were fit to 1:1 (top), 1:2 (middle) and 2:1 (bottom) binding models. Proton 1 (N*H*; starting at 9.71 ppm) and proton 2 (N*H*; starting at 9.12 ppm) were monitored.

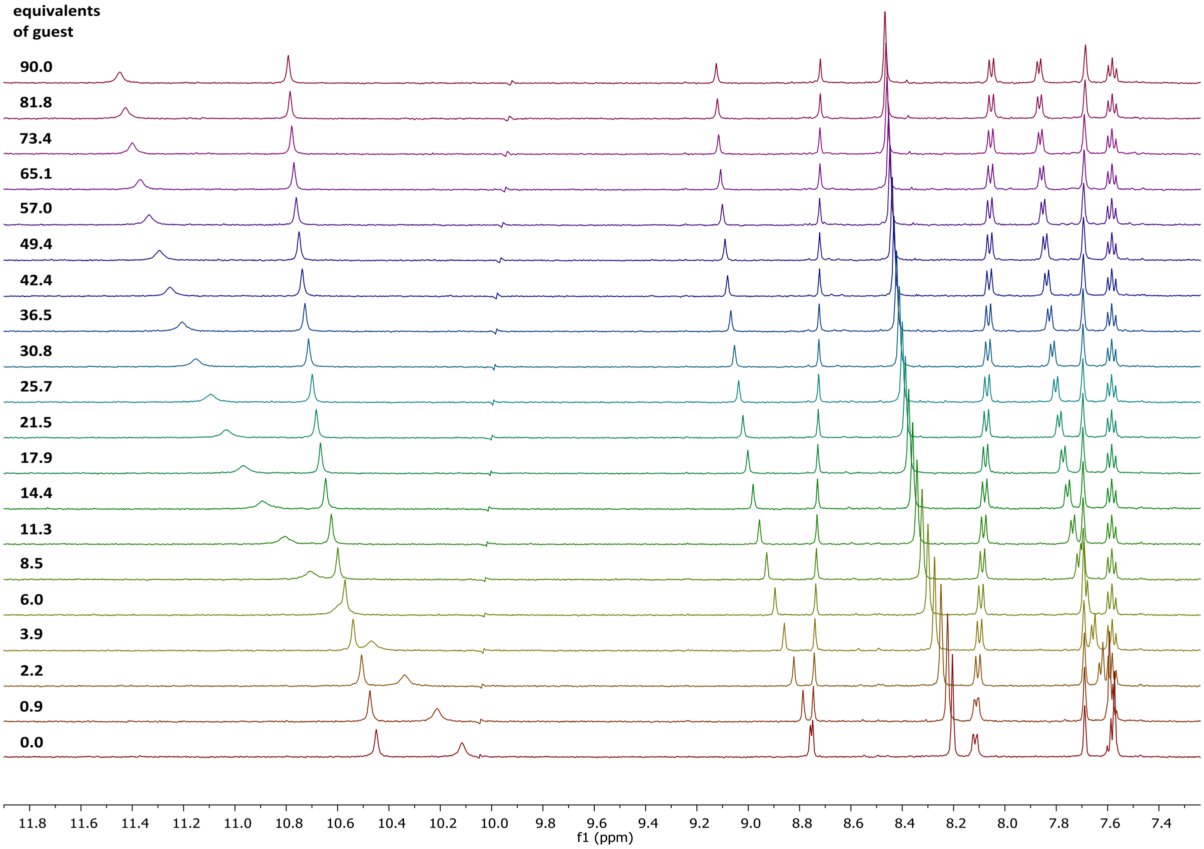

Figure S28. Downfield region of the ^1H NMR spectra for the titration of $Bu_4N^+Cl^-$ into **7SF2** in DMSO-d_6/0.5% H_2O at 298 K.

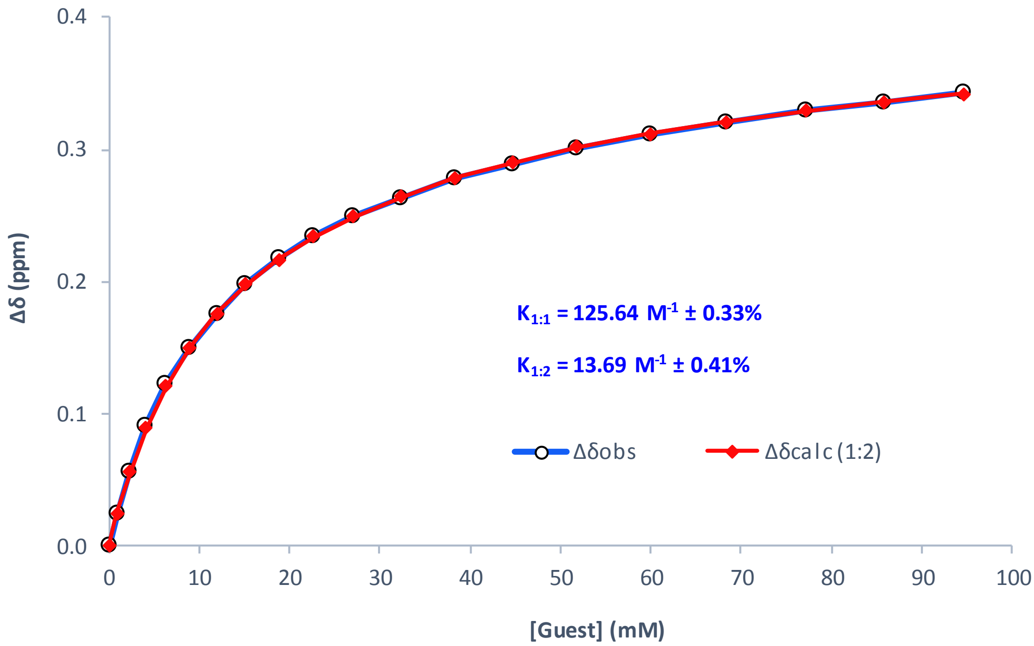

Figure S29. Observed binding curve (N*H*; starting at 10.45 ppm) and calculated fit (1:2 binding model) for **7SF2** when titrated against $Bu_4N^+Cl^-$ in DMSO-d_6/0.5% H_2O at 298 K.

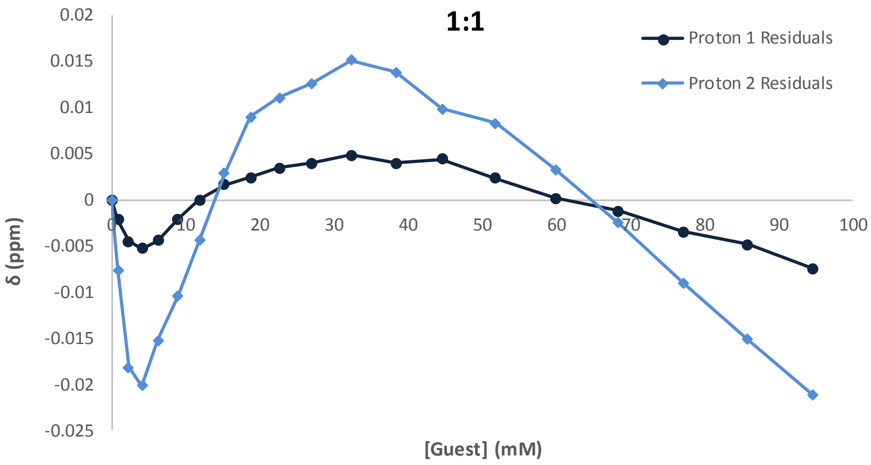

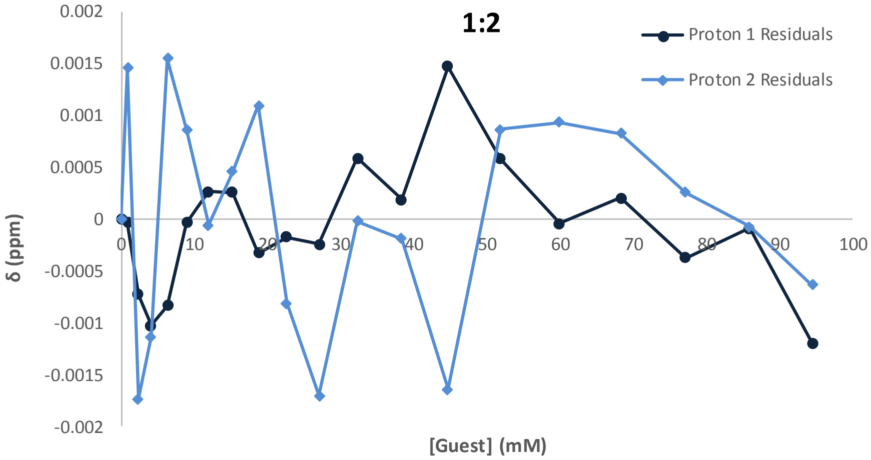

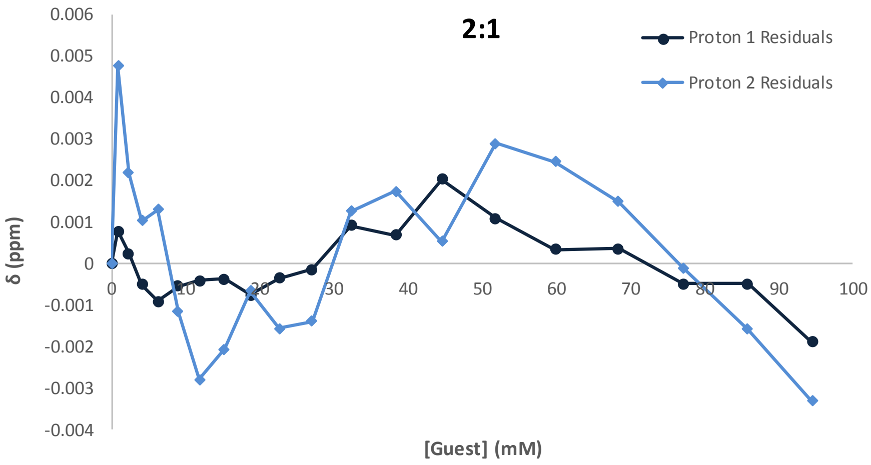

Figure S30. Residual distribution plots for **7SF2** when titration data were fit to 1:1 (top), 1:2 (middle) and 2:1 (bottom) binding models. Proton 1 (N*H*; starting at 10.45 ppm) and proton 2 (N*H*; starting at 10.11 ppm) were monitored.

4. Transport Studies

4.1. General Considerations

1-palmitoyl-2-oleoyl-*sn*-glycero-3-phosphocholine (POPC) and 1,2-dipalmitoyl-*sn*-glycero-3-phosphocholine (DPPC) were obtained from Avanti® Polar Lipids, Inc. Cholesterol was obtained from Aldrich Chem. Co. Deacidified $CHCl_3$ was obtained by passing $CHCl_3$ through a column of activated basic alumina. All aqueous solutions were prepared using deionised water that had been passed through a Millipore filtration system.

Transport studies were conducted using a PerkinElmer LS 45 fluorescence spectrometer, exciting the sample at 450 nm and measuring the emission at 535 nm. Samples were stirred vigorously during the experiment. Quartz cuvettes (4 windows, 10 mm pathlength) were used for all optical measurements.

4.2. The Lucigenin Method

Stock solutions of POPC (10-15 mM) and cholesterol (7-9 mM) in deacidified $CHCl_3$ were prepared. For measurements at a transporter to lipid loading of 1:25k, a stock solution of the transporter (**T1**) was prepared by dissolving the transporter (0.5-1 mg) in 25 mL of methanol, acetone or DMSO (depending on its solubility). For measurements at loadings of 1:50k and below, a new stock solution (**T2**) was prepared by transferring 100 µL of solution **T1** to a volumetric flask and diluting to 10 mL with the solvent used previously.

Solutions of POPC (4.20 µmol) and cholesterol (1.80 µmol) were added to a 5 mL round bottom flask (7:3 ratio) followed by a calculated amount of either solution **T1** or **T2** to give the desired transporter to lipid loading. The volatile solvents were removed under a stream of nitrogen[‡] and the resultant lipid film dried under high vacuum (1 h). To the flask was added a solution of lucigenin (0.8 mM, 500 µL) in aqueous $NaNO_3$ (225 mM) and the mixture was sonicated (30 s). The resultant yellow suspension was stirred vigorously (1 h) and then subjected to ten freeze-thaw cycles (flask placed in liquid nitrogen and then in warm water). The vesicle suspension was then transferred to a sizing apparatus and extruded through a polycarbonate membrane (200 nm pores) twenty-nine times. The resized vesicles were loaded onto a size exclusion column (packed with Sephadex® G-50 resin) and eluted with aqueous $NaNO_3$ solution (225 mM) to separate the vesicles from external lucigenin. The first cloudy

[‡] When DMSO was used to prepare the transporter stock solution, the round bottom flask was either left under high vacuum overnight or freeze-dried for ~2 h to ensure the complete removal of this solvent.

band was collected and diluted with aqueous $NaNO_3$ solution (225 mM) to give a total volume of 15 mL (0.4 mM final lipid concentration).

To a cuvette equipped with a magnetic stirrer, was added the suspension of vesicles (3 mL) and placed inside the fluorescence spectrometer (temperature maintained at 25 °C). After the signal had stabilised (~90 s), the measurement was initiated. After 30 s, a solution of NaCl (1 M, 75 µL) in aqueous $NaNO_3$ (225 mM) was added to the cuvette and the measurement allowed to run for 11 min. This process was repeated a further three times.

Data were processed using Origin 9 (Academic): for each run, the initial plateau (before the addition of chloride) was removed. The data was then normalised by dividing all fluorescence values (F) by the maximum fluorescence value (F_0) for the individual runs. From the mean average of this data, the vertical drop (the first 1-2.5 s after chloride addition due to quenching of external lucigenin) was removed. The data were then normalised to give a final trace of relative fluorescence (F/F_0) versus time.

4.3. Quantification of Transport Rates

As described in our previous publication,[8] the inverse of the normalised fluorescence trace (F_0/F) is directly proportional to the concentration of chloride inside the vesicles (according to the Stern-Volmer equation). Thus F_0/F was used in the quantification of transport rates.

Half-life ($t_{½}$): Determined by fitting the F_0/F curve (0-500 s) to a single exponential decay function:

$$F_0/F = y - a\mathrm{e}^{-bt}$$

Obtaining a value for the fit parameter *b* allows for the half-life ($t_{½}$) to be calculated:

$$t_{½} = \ln(2)/b$$

Initial Rate (*I*): Determined by fitting the F_0/F curve (0-500 s) to a double exponential decay function:

$$F_0/F = y - a\mathrm{e}^{-bt} - c\mathrm{e}^{-dt}$$

Differentiating at $t = 0$ allows for the initial rate (*I*) to be calculated:

$$I = (\mathrm{a} \times \mathrm{b}) + (\mathrm{c} \times \mathrm{d})$$

Specific Initial Rate ([*I*]): Initial rate (*I*) divided by transporter/lipid ratio and averaged for a range of experiments at different loadings. Since the value obtained is independent of the transporter to lipid loading, it allows the performance of different anion transporters to be compared directly.

4.4. Hill Analysis on Bisurea 7ON

Hill analysis was applied to bisurea **7ON** to calculate an $EC_{50,\ 270\ s}$ value. In our case this refers to the amount of transporter required to give an intravesicular chloride concentration of 12.5 mM, 270 s after the addition of NaCl in the lucigenin assay.

Using F_0/F values at 270 s as a measure of chloride concentration inside the vesicles at that time, these values were plotted against the corresponding concentration of transporter used for seven different loadings of transporter. The data were then fitted with the Hill1 equation in Origin 2016 (Figure S31) to give an $EC_{50,\ 270\ s}$ value of 3.0×10^{-6} (or 0.0003 mol%) as represented by the parameter *k*.

Hill analysis also allows for the Hill coefficient (*n*) to be determined, which indicates how many molecules of receptor form the active transporting complex; this fit gave a value of n = 1.05.

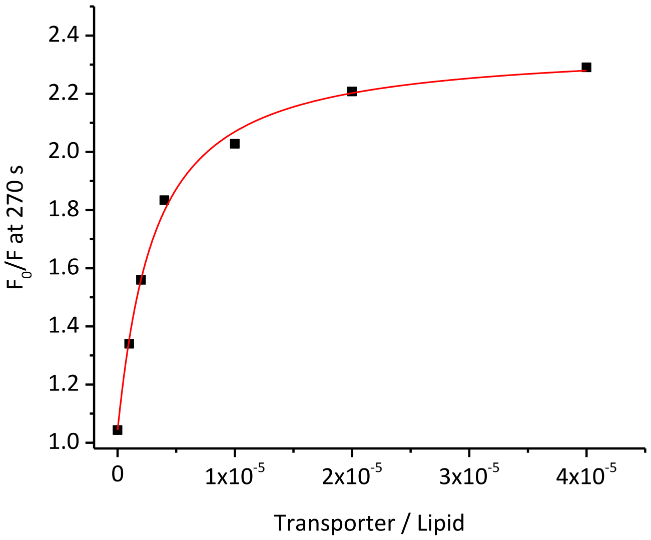

Model	Hill1
Equation	y = START + (END - START) * x^n / (k^n + x^n)
Plot	F0/F @ 270 s
START	1.0432 ± 0
END	2.36141 ± 0.05062
k	3.03262E-6 ± 3.24584E-7
n	1.05202 ± 0.10536
Reduced Chi-Sqr	8.59191E-4
R-Square(COD)	0.99733
Adj. R-Square	0.996

Figure S31. Plot of F_0/F (representative of intravesicular chloride concentration) 270 s after the addition of NaCl versus the concentration of **7ON** inside the vesicles. The equation used for the fit is shown in the table. Parameters *k* and *n* correspond to the $EC_{50,\ 270\ s}$ value and Hill coefficient respectively.

4.5. Deliverability Studies

Stock solutions of previously reported anionophores were prepared using HPLC grade methanol at either 96 µM (for **1a**) or 9.6 µM (for **1b** and **2b**).[9,10] However, due to the low solubility of the anthracene bisureas in methanol, solutions of these compounds were prepared using DMF instead at either 9.6 µM (for **7OP**) or 0.96 µM (for **7ON**, **7OF** and **7OF2**).

LUVs were prepared in the same manner as described in Section 4.2, except that the anionophore was not added to the initial lipid mixture. After size exclusion chromatography, the collected vesicles were diluted with aqueous $NaNO_3$ (225 mM) to give a suspension with a notional lipid concentration of 0.4 mM and 3 mL of this was transferred to a cuvette. The anionophore solution (5 µL) was added externally to the cuvette by rapid plunger action of a 10 µL syringe with the tip of the needle positioned just above the stirrer. Assuming the notional lipid concentration of 0.4 mM, this would give a transporter to lipid loading of 1:2500 (for **1a**), 1:25k (for **1b**, **2b** and **7OP**) and 1:250k (for **7ON**, **7OF** and **7OF2**). NaCl (1 M, 75 µL) in aqueous $NaNO_3$ (225 mM) was added 5 min after the addition of anionophore and the measurement allowed to run for a further 11 min. This process was repeated twice more, and data were processed as before. The corresponding F/F_0 traces are shown in Figure 8 of the main text for anthracenes **7**, and have been previously reported for cholapods **1a** and **1b** (Ref. 9) and decalin **2b** (Ref. 10).

Deliverability (*D*) was calculated by dividing the initial rate (*I*) of transport from this experiment by that measured when the anionophore was preincorporated. Values obtained for *D* are reported in Table 1 of the main text.

4.6. Transport Mediated by 7ON in DPPC Vesicles

To help elucidate the mechanism by which **7ON** mediates anion transport, experiments were performed using vesicles composed of DPPC. This lipid undergoes a gel–liquid phase transition at 41 °C.[11] If an anionophore is operating via a channel mechanism, then similar rates of transport would be expected at 25 °C (gel phase) and 45 °C (liquid phase), since channel-mediated transport is relatively insensitive to membrane viscosity. Conversely, if an anionophore is acting as a mobile carrier, then a significant attenuation in activity would be expected below 41 °C, since the more viscous membrane hinders diffusion of a carrier.[11,12]

Experimental

To a 5 mL round bottom flask was added DPPC (516 µL, 11.62 mM, 6.0 µmol) in deacidified $CHCl_3$ followed by **7ON** (137 µL, 437 nM, 60 pmol) in DMSO (1:100k transporter to lipid loading). The volatile solvents were removed under a stream of nitrogen. To ensure the complete removal of DMSO, the mixture was freeze-dried overnight. The lipid film was hydrated with lucigenin (0.8 mM, 500 µL) in aqueous $NaNO_3$ (225 mM), sonicated for 30 min at 40 °C and then stirred vigorously for 1 h at 50 °C. After this time the vesicles were subjected to ten freeze-thaw cycles (flask placed in liquid nitrogen and then in warm water) and then extruded through a polycarbonate membrane (200 nm pores) twenty-nine times, ensuring the temperature was kept at 50 °C. The resized vesicles were loaded onto

a size exclusion column (packed with Sephadex® G-50 resin) and eluted with aqueous $NaNO_3$ solution (225 mM) to separate the vesicles from external lucigenin. The first cloudy band was collected and diluted with aqueous $NaNO_3$ solution (225 mM) to give a total volume of 15 mL (0.4 mM final lipid concentration).

To a cuvette equipped with a magnetic stirrer was added the suspension of vesicles (3 mL). The cuvette was placed inside the fluorescence spectrometer with the temperature maintained at either 25 °C or 45 °C. After the signal had stabilised, the measurement was initiated. After 30 s, a solution of NaCl (1 M, 75 µL) in aqueous $NaNO_3$ (225 mM) was added to the cuvette and the measurement allowed to run for a further 11 min. This experiment was conducted twice at each temperature with the data processed as before (see Section 4.2). The F/F_0 traces are shown in Figure S32. The considerable difference in activity observed at 25 °C (gel phase) and 45 °C (liquid phase) supports a mobile carrier mechanism.

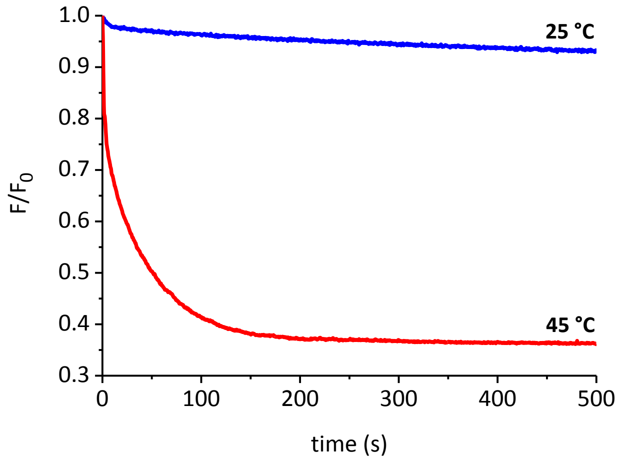

Figure S32. Chloride transport mediated by **7ON** in DPPC vesicles at 25 °C (gel phase) and 45 °C (liquid phase) when preincorporated at a 1:100k transporter to lipid loading.

Regen and co-workers have reported an issue with this method in that an ionophore can be expelled from DPPC vesicles when the lipid is in the gel phase.[13] Thus a lower activity observed at 25 °C compared to 45 °C may not necessarily indicate a mobile carrier mechanism, but instead reflect a lower concentration of transporter present in the membrane at 25 °C. Consequently, even if an ionophore functions via a channel mechanism, it may still display lower activity at temperatures below 41 °C if it is ejected from the membrane. However, in the case of bisurea **7ON**, this molecule is lipophilic enough that it should not be expelled from the vesicles. In support of this is that in experiments employing POPC/cholesterol vesicles, we find that **7ON** resides exclusively within the membrane and is not capable of leaching into the aqueous phase (see Section 4.9). Furthermore, the procedure used to prepare DPPC vesicles involves size exclusion chromatography (*vide supra*), which

is performed at room temperature. Thus, if an anionophore were expelled at 25 °C, it would have already been removed before the measurement of chloride transport had commenced. Consequently, no observable activity would be expected even when the lipid is in the liquid phase. However, the significant activity observed at 45 °C indicates that **7ON** is not ejected from the membrane. It is therefore reasonable to apply the conventional interpretation of this experiment.

4.7. Exchange of Bisurea 7ON between Vesicles

To determine if **7ON** is capable of exchanging between vesicles, an experiment was performed where "delivery vesicles" containing **7ON** but not lucigenin were mixed with "receiver vesicles" containing lucigenin but not **7ON**.

Delivery vesicles (1:25k transporter to lipid loading) were prepared from POPC (91 µL, 15.46 mM, 1.40 µmol), cholesterol (89 µL, 6.78 mM, 0.60 µmol) and **7ON** (183 µL, 437 nM, 80 pmol) in a manner analogous to that described in Section 4.2. However, these vesicles were hydrated with aqueous $NaNO_3$ (225 mM) instead of lucigenin; consequently size exclusion chromatography was not required. After the delivery vesicles were sized, they were diluted with aqueous $NaNO_3$ (5 mL, 225 mM) to give a final lipid concentration of 0.4 mM.

Receiver vesicles were prepared from POPC (453 µL, 15.46 mM, 7.0 µmol) and cholesterol (443 µL, 6.78 mM, 3.0 µmol) in a manner analogous to that described in Section 4.2. However, the anionophore was not added to the initial lipid mixture. After size exclusion chromatography, the receiver vesicles were diluted with aqueous $NaNO_3$ (25 mL, 225 mM) to give a final lipid concentration of 0.4 mM.

The delivery and receiver vesicles were combined in a 1:9 ratio to give a notional transporter to lipid loading of 1:250k. The mixture was stirred for a specified amount of time before the addition of NaCl (1 M, 75 µL) in aqueous $NaNO_3$ (225 mM) to 3 mL of the vesicle suspension. Data from the fluorescence output traces was processed as before and is shown in Figure S33.

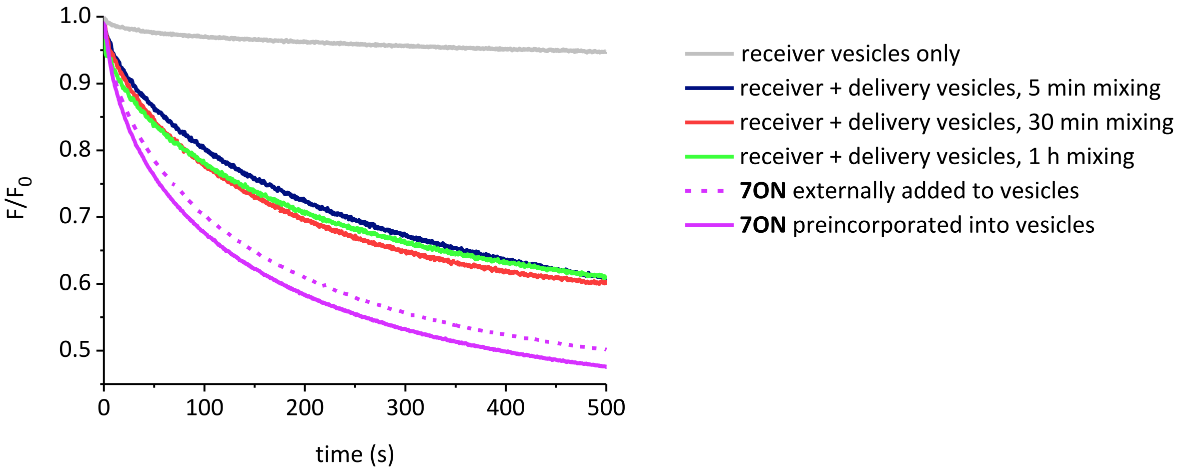

Figure S33. Chloride transport into vesicles which initially contained no transporter as measured by the quenching of lucigenin fluorescence: delivery vesicles (containing **7ON** but not lucigenin) were stirred with receiver vesicles (containing lucigenin but not **7ON**) in a 1:9 ratio for a specified amount of time before NaCl was added. Traces obtained for when **7ON** was either preincorporated into vesicles containing lucigenin or added externally as a solution in DMF have been included for comparison. The notional transporter to lipid ratio was 1:250k for all experiments.

After stirring the vesicles for 5 minutes, a decay in fluorescence is observed, which indicates that chloride is being transported into vesicles that were originally prepared without transporter. This result is consistent with the transfer of **7ON** from delivery to receiver vesicles and thus proves that **7ON** is capable of exchanging between vesicles. Stirring the vesicles for more than 5 minutes does not affect the rate of chloride transport, which indicates that the exchange of **7ON** between vesicles is fast (< 5 min) relative to the timescale of the transport measurement (11 min).

4.8. Exchange of Bisurea 7OF2 between Vesicles

To determine if **7OF2** is capable of exchanging between vesicles, an experiment analogous to that described in Section 4.7 was performed. However, in this case, a higher loading of transporter **7OF2** (14.77 µL, 54.17 µM, 0.80 nmol) was used when preparing the delivery vesicles to give a 1:2500 transporter to lipid loading. Mixing the delivery and receiver vesicles in a 1:9 ratio gives a final, notional transporter to lipid loading of 1:25k. Data from the fluorescence output traces was processed as before and is shown in Figure S34.

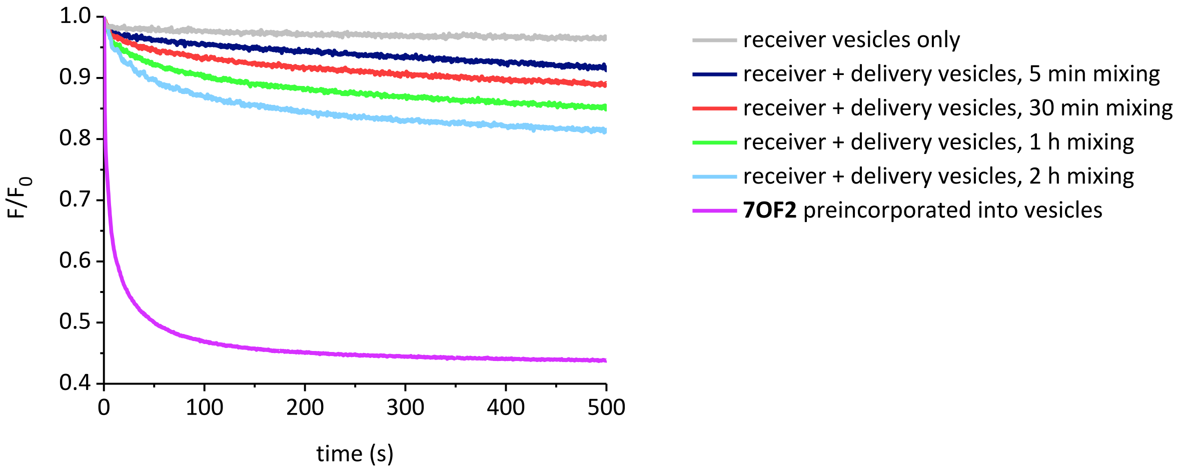

Figure S34. Chloride transport into vesicles which initially contained no transporter as measured by the quenching of lucigenin fluorescence: delivery vesicles (containing **7OF2** but not lucigenin) were stirred with receiver vesicles (containing lucigenin but not **7OF2**) in a 1:9 ratio for a specified amount of time before NaCl was added. The trace obtained for when **7OF2** was preincorporated into vesicles containing lucigenin has been included for comparison. The notional transporter to lipid ratio was 1:25k for all experiments.

After stirring the vesicles for 5 minutes, only a slight decay in fluorescence is observed, which indicates that most of the transporter is still confined to the delivery vesicles. The decay in fluorescence becomes more pronounced as the vesicles are mixed for longer periods of time, consistent with the receiver vesicles becoming more populated with transporter over time. If a full equilibrium would be reached, where **7OF2** is distributed evenly over all vesicles, then the final transporter to lipid loading would be 1:25k and the corresponding fluorescence trace should be identical to that shown in Figure S34 for the preincorporated transporter. However, the considerable difference between this trace and that obtained after mixing the vesicles for 2 hours, would indicate that even after 2 hours, the distribution of **7OF2** amongst all vesicles is still very uneven. We therefore conclude that although **7OF2** can exchange between vesicles, this process is very slow (> 2 hours) relative to the timescale of the transport measurement (11 min).

4.9. Leaching Studies on Bisurea 7ON

To determine whether **7ON** resides exclusively within the membrane of vesicles, or if an equilibrium exists where some of the transporter is present in the aqueous phase, a leaching study was performed.

Solutions of POPC (7 µmol) and cholesterol (3 µmol) were added to a 5 mL round bottom flask (7:3 ratio) followed by a solution of the transporter (40 pmol) to give a 1:250k transporter to lipid loading.

Thereafter the procedure as outlined in Section 4.2 was followed to form LUVs (~200 nm). After size exclusion chromatography, the vesicles were collected in a 5 mL volumetric flask to give a notional lipid concentration of 2.0 mM. Successive dilutions with aqueous $NaNO_3$ (225 mM) provided suspensions with lipid concentrations of 0.4 mM, 0.2 mM and 0.1 mM. All vesicles suspensions were stored on ice until required. The experiment was commenced by the addition NaCl (1 M, 75 µL) in aqueous $NaNO_3$ (225 mM) to 3 mL of the vesicle suspension. For each lipid concentration, 2-3 replicate experiments were performed and the averaged fluorescence decay traces are shown in Figure S35.

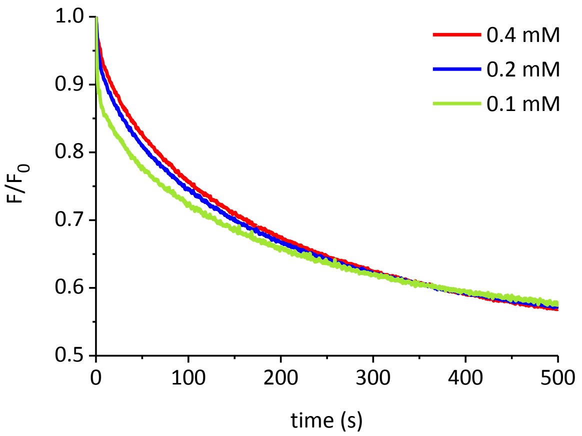

Figure S35. Chloride transport mediated by **7ON** in 200 nm POPC/cholesterol (7:3) LUVs as measured using the lucigenin method. The total lipid concentration was varied from 0.4 to 0.1 mM.

If a transporter is capable of leaching from the membrane into the aqueous phase, then dilution of the vesicles is expected to lower the concentration of transporter present in the lipid bilayers as more of it partitions into the aqueous phase. Consequently, an attenuation in activity is expected at lower lipid concentrations.[14] The traces presented in Figure S35 are all roughly superimposable, thus dilution does not affect observed activity. We therefore conclude that **7ON** resides solely within the membrane and is not capable of leaching.

5. Decomposition Studies

As described in the main text, bisthiourea **7SF2** was found to be significantly less active than its bisurea analogue (**7OF2**) and indeed all other anthracene-based bisureas tested. We postulated that this result might indicate that **7SF2** is unstable in methanol (the solvent used to prepare a stock solution of this compound for use in the lucigenin assay). We thus investigated this further; a fresh stock solution of **7SF2** (35 µM, 25 mL) in methanol was prepared. After 2 h, the solvent was removed *in vacuo* and the residue analysed by ^{1}H and ^{19}F NMR spectroscopy (Figures S36 and S37 respectively).

From these spectra, it is evident that decomposition of **7SF2** has occurred in methanol and this is likely a major cause of the relatively low activity observed in the lucigenin assay.

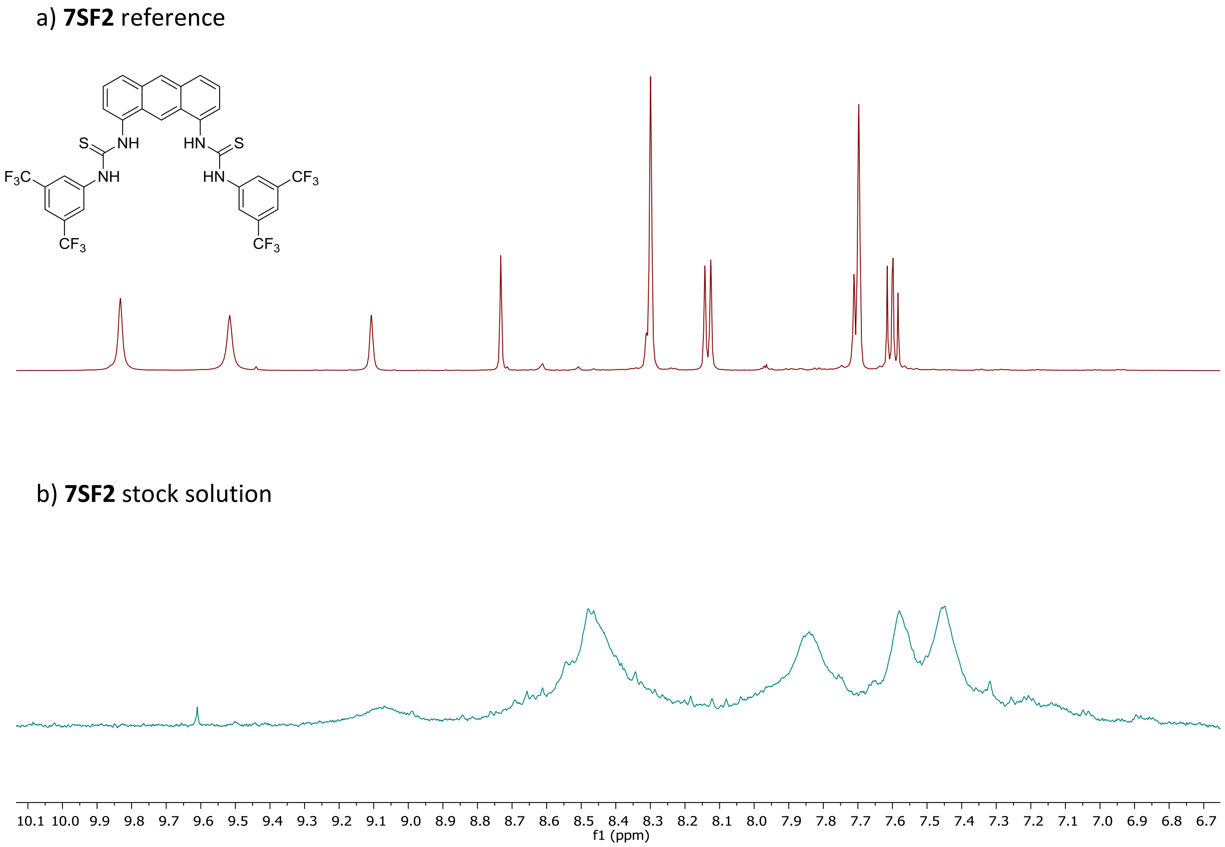

Figure S36. Stacked ^{1}H NMR spectra for **7SF2** recorded in acetone-d_6: a) reference spectrum; b) spectrum of the residue obtained after a solution of **7SF2** in methanol was concentrated *in vacuo* after 2 h.

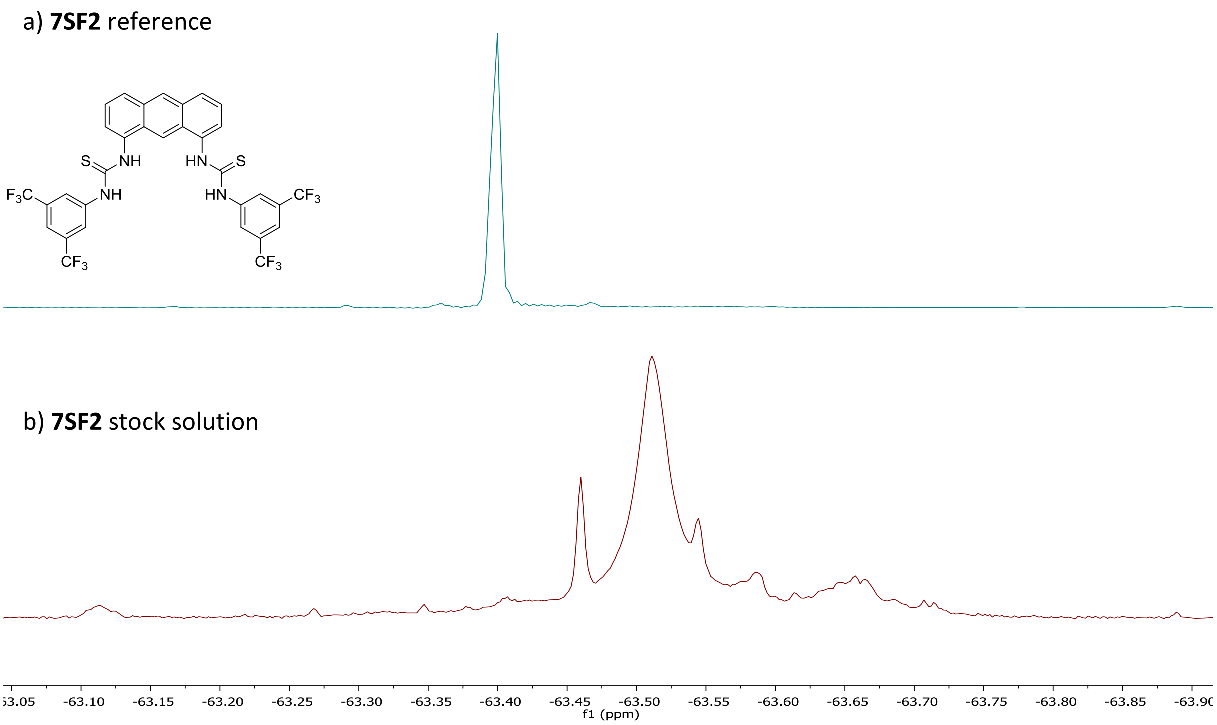

Figure S37. Stacked ^{19}F NMR spectra for **7SF2** recorded in acetone-d_6: a) reference spectrum; b) spectrum of the residue obtained after a solution of **7SF2** in methanol was concentrated *in vacuo* after 2 h.

6. References

[1] A. B. Pangborn, M. A. Giardello, R. H. Grubbs, R. K. Rosen and F. J. Timmers, *Organometallics*, 1996, **15**, 1518-1520.

[2] A. Dahan, T. Ashkenazi, V. Kuznetsov, S. Makievski, E. Drug, L. Fadeev, M. Bramson, S. Schokoroy, E. Rozenshine-Kemelmakher and M. Gozin, *J. Org. Chem.*, 2007, **72**, 2289-2296.

[3] K. Kellett, J. H. Broome, M. Zloh, S. B. Kirton, S. Fergus, U. Gerhard, J. L. Stair and K. J. Wallace, *Chem. Commun.*, 2016, **52**, 7474-7477.

[4] J. Y. Kwon, Y. J. Jang, S. K. Kim, K.-H. Lee, J. S. Kim and J. Yoon, *J. Org. Chem.*, 2004, **69**, 5155-5157.

[5] F. Ulatowski, K. Dąbrowa, T. Bałakier and J. Jurczak, *J. Org. Chem.*, 2016, **81**, 1746-1756.

[6] P. Thordarson, *Chem. Soc. Rev.*, 2011, **40**, 1305-1323.

[7] D. Brynn Hibbert and P. Thordarson, *Chem. Commun.*, 2016, **52**, 12792-12805.

[8] H. Valkenier, L. W. Judd, H. Li, S. Hussain, D. N. Sheppard and A. P. Davis, *J. Am. Chem. Soc.*, 2014, **136**, 12507-12512.

[9] H. Li, H. Valkenier, L. W. Judd, P. R. Brotherhood, S. Hussain, J. A. Cooper, O. Jurček, H. A. Sparkes, D. N. Sheppard and A. P. Davis, *Nat. Chem.*, 2016, **8**, 24-32.

[10] N. López Mora, A. Bahreman, H. Valkenier, H. Li, T. H. Sharp, D. N. Sheppard, A. P. Davis and A. Kros, *Chem. Sci.*, 2016, **7**, 1768-1772.

[11] M. J. Pregel, L. Jullien, J. Canceill, L. Lacombe and J.-M. Lehn, *J. Chem. Soc., Perkin Trans. 2*, 1995, **0**, 417-426.

[12] G. Deng, T. Dewa and S. L. Regen, *J. Am. Chem. Soc.*, 1996, **118**, 8975-8976.

[13] S. Otto, M. Osifchin and S. L. Regen, *J. Am. Chem. Soc.*, 1999, **121**, 10440-10441.

[14] H. Valkenier, C. J. E. Haynes, J. Herniman, P. A. Gale and A. P. Davis, *Chem. Sci.*, 2014, **5**, 1128-1134.
